# Microplastics and Oxidative Stress—Current Problems and Prospects

**DOI:** 10.3390/antiox13050579

**Published:** 2024-05-08

**Authors:** Kornelia Kadac-Czapska, Justyna Ośko, Eliza Knez, Małgorzata Grembecka

**Affiliations:** Department of Bromatology, Faculty of Pharmacy, Medical University of Gdańsk, 80-416 Gdańsk, Poland; kornelia.kadac@gumed.edu.pl (K.K.-C.); justyna.osko@gumed.edu.pl (J.O.); eliza.knez@gumed.edu.pl (E.K.)

**Keywords:** microplastics, plastic, oxidative stress, reactive oxygen species, human health risk

## Abstract

Microplastics (MPs) are plastic particles between 0.1 and 5000 µm in size that have attracted considerable attention from the scientific community and the general public, as they threaten the environment. Microplastics contribute to various harmful effects, including lipid peroxidation, DNA damage, activation of mitogen-activated protein kinase pathways, cell membrane breakages, mitochondrial dysfunction, lysosomal defects, inflammation, and apoptosis. They affect cells, tissues, organs, and overall health, potentially contributing to conditions like cancer and cardiovascular disease. They pose a significant danger due to their widespread occurrence in food. In recent years, information has emerged indicating that MPs can cause oxidative stress (OS), a known factor in accelerating the aging of organisms. This comprehensive evaluation exposed notable variability in the reported connection between MPs and OS. This work aims to provide a critical review of whether the harmfulness of plastic particles that constitute environmental contaminants may result from OS through a comprehensive analysis of recent research and existing scientific literature, as well as an assessment of the characteristics of MPs causing OS. Additionally, the article covers the analytical methodology used in this field. The conclusions of this review point to the necessity for further research into the effects of MPs on OS.

## 1. Introduction

Microplastics (MPs) are plastic particles with sizes ranging from 0.1 to 5000 µm [1]. The capacity of MPs to pass through biological barriers while maintaining a high surface-area-to-mass ratio and the potential for accumulation in higher-trophic-level organisms through the food chain are both significant [2]. Due to their pervasiveness and potential effects on the environment and public health, these particles are a global concern. Therefore, scientists urgently need to learn more about the sources, characteristics, ecological effects, and health effects of MPs.

Microplastics can be found in both aquatic and terrestrial environments. They enter ecosystems through improper waste disposal, industrial discharges, and sewage, and atmospheric transport causes their deposition even in the most remote regions of the planet. Plastic fragments can be found in soil, air, freshwater, and saltwater tanks, as well as in food. This demonstrates how widespread this kind of contamination is [3].

Microplastics can be classified into primary and secondary groups based on their origin. The first category consists of particles that have been purposefully created by humans and added to particular goods, e.g., face scrubs [4]. The degradation and fragmentation of plastic objects lead to the formation of the second group. The degradation process can aggravate the toxicity of MPs due to their increased oxidative potential and decreased particle size. For instance, IL-1β levels in response to MPs showed dependency on the size of the particles (1 µm–10 µm), where the highest level was observed after treatment with 1 µm particles. Treatment doses of MPs were in the range 62.5–250 µg/mL and showed an increase in the level of IL-1β proportional to the dose [5]. The majority of MPs’ contribution to environmental contamination comes from these two processes [6]. Both in abiotic matrices and in living things, plastic particles can break up [6]. Microplastics can enter the human body through ingestion, inhalation, or dermal contact [7,8]. The occurrence of plastic particles in the human organism, e.g., blood samples, has been confirmed [9]. However, the direct impact of MPs on human health has not yet been proven.

The main components of MPs are polymers, including polyethylene (PE), polypropylene (PP), poly(ethylene terephthalate) (PET), and polystyrene (PS). These types of particles exhibit different sizes, shapes, and colors [6]. Concerns about MPs relate to the type of plastic, the harmfulness of the constituent polymers, the potential release of additives introduced during the processing of the polymer material, and the physical presence of plastic fragments (particle size and surface functionalization). Microplastics’ capacity to adsorb metals and organic compounds poses a threat [10]. Microplastics have the potential to transport different contaminants between various ecosystems. The negative effects of MPs on a range of organs and functions may therefore be exacerbated by concurrent exposure to additional substances that have been adsorbed on the surfaces of MPs [11]. Particularly, the toxicity brought on by MPs in combination with additives like bisphenols, phthalates, and persistent organic pollutants (POPs) is greater than the toxicity induced by MPs alone [12,13,14]. Additionally, biofilms, which are bacteria that colonize plastic particles’ surfaces, might facilitate the adsorption of pollutants [15]. Furthermore, environmental conditions, exposure time, route, and concentration all have an impact on their harmfulness [15]. However, there is currently a lack of systematic knowledge regarding MPs’ impact on health. This leaves a sizable knowledge gap regarding the dangers posed by plastic particles.

Numerous studies suggest that oxidative stress (OS) may play a role in the harm caused by MPs [2,16]. The concept of OS in toxicology involves not only the initial oxidative effects caused by particles but also the subsequent generation of reactive oxygen species (ROS) in cells or tissues exposed to them. When plastic particles are absorbed, the integrity of the cell membrane is compromised, the lipid bilayer is altered, pores form, and the production of intracellular ROS is increased. In turn, the generation of ROS leads to mitochondrial dysfunction, the release of pro-inflammatory cytokines, and cell damage [17,18,19].

The idea of MPs as a determinant for OS has garnered significant attention and has been associated with various aspects of particle toxicology. Thus, the purpose of this work is to provide a critical review of whether the harmfulness of MPs that constitute environmental contaminants may result from OS by correlating plastic particles with observed biological effects in cells, tissues, organs, and whole organisms. Exploring the relationship between these ostensibly unrelated concepts will help us better understand whether MPs pose a threat to the environment and living things. In-depth comprehension of the described issue will be possible through a review of the already-published scientific literature and the investigation of various aspects of the presence of plastic particles in the environment and their potential to cause OS. In addition, this review also discusses research methodology on the subject, identifies knowledge gaps, and offers recommendations to improve future analysis. In order to lessen the effects of the escalating environmental crisis triggered by the presence of MPs, this article emphasizes the urgent need for interdisciplinary research and mitigation strategies.

## 2. Materials and Methods

During the preparation of the presented review, scientific articles that were published after 2018 from PubMed, ScienceDirect, and Scopus databases were analyzed. These were peer-reviewed papers written in English. Older articles were included occasionally, only when they made a significant contribution to the field of knowledge described. “Microplastics” in combination with the terms “oxidative stress” or “reactive oxygen” in the title, keywords, or abstract were used as search terms. The search “microplastics” and “oxidative stress” retrieved 718 publications, while the search “microplastics” and “reactive oxygen” allowed for the retrieval of 253 articles. The results were subsequently refined by incorporating names of polymers that are common environmental contaminants: “polystyrene”, “polyethylene”, “poly(vinyl chloride)”, “polytetrafluoroethylene”, and “poly(lactic acid)” or phrases like “signaling pathway”, “lipid peroxidation”, “DNA damage”, “enzyme”, “cell damage”, “cell membrane”, “lysosomes”, “mitochondria”, “endoplasmic reticulum”, “tissue”, “inflammation”, “reproduction”, “rat”, and “mouse”. After that, the texts of the articles were analyzed, and the basic information was summarized. The primary criteria employed for paper selection were that they needed to be related to the following:

(1) Plastic particles, which are common environmental contaminants and exist in the size characteristic of MPs (from 0.1 to 5000 µm);
(2) The evaluation of the direct and indirect impacts of oxidative stress in humans and animals.

Studies failing to meet these criteria were excluded. In preparing this article, we tried to include a complete characterization of plastic particles. Papers that did not include the names and precise sizes of MPs were excluded from this review. The analysis of 194 scientific papers was carried out.

## 3. Results and Discussion

### 3.1. Oxidative Stress: Mechanisms and Implications

Oxidative stress is “an imbalance between oxidants and antioxidants in favor of the oxidants, leading to a disruption of redox signaling and control and/or molecular damage” [20,21,22]. It is influenced by both internal and external factors, such as the health status and age of the exposed person, UV radiation, cigarette smoke, air pollution, or diet. When ROS are generated via natural body functioning, this process can be called oxidative eustress. Oxidative stress involving a pathological imbalance between the production and scavenging of free radicals is called distress [21,23,24]. Thus, we can refer to MP-induced oxidative stress as distress.

#### 3.1.1. Mechanisms of ROS Generation

**Figure 1:**
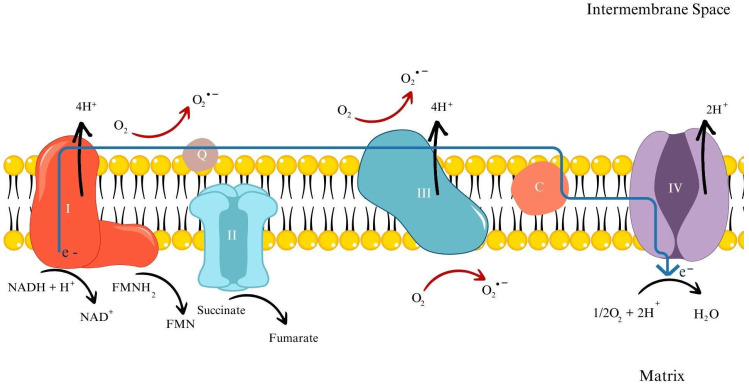
Simplified diagram of mitochondrial respiratory chain. Arrows indicate direction of changes during the process. Red arrows show generation of reactive oxygen species, black arrow indicate transformations and reactions of substrates, and blue arrow indicate direction of electrons flow. Subsequent mitochondrial complexes are marked by Roman numbers; Q—coenzyme Q_10_ and C—cytochrome C.

Over the course of evolution, organisms have developed an effective process for producing energy in the form of adenosine triphosphate (ATP) using oxygen. This mechanism is the electron transport chain (ETC) conducted in the inner mitochondrial membrane and results in the generation of ATP and ROS [25]. Radicals that exhibit the biggest health implications are superoxide anion radical (O_2_^●−^), hydroxyl radical (^●^OH), and hydrogen peroxide (H_2_O_2_). The superoxide anion radical is the major ROS produced in the ETC [26] (Figure 1).

In the organism, the second most common mechanism generating ROS is the oxidation of reduced forms of low-molecular-weight cellular components (RH_2_). In the presence of oxygen (O_2_), they undergo one-electron oxidation with the production of O_2_^●−^ and a free radical:

RH_2_ + O_2_ → ^●^RH + H^+^ + O_2_^●−^

For example, glutathione, cysteine, catecholamines, and reduced flavine nucleotides (FMNH_2,_ FADH_2_) are subjected to these reactions.

Another mechanism responsible for ROS generation is the oxidation of respiratory proteins (RP), or hemoproteins. These are hemoglobin and myoglobin. This occurs due to the presence of iron (Fe) in the complexes with the compounds mentioned. Respiratory proteins are capable of fulfilling their function only if Fe is in the form of Fe^2+^. However, Fe^2+^ easily undergoes oxidation to Fe^3+^ with the generation of O_2_^●−^ [27]:

RP—Fe^2+^—O_2_ → RP—Fe^3+^—O_2_^●−^

It was proven that ^●^OH has the most destructive influence on organisms and viable cells. The source of this radical in vivo is the Haber–Weiss reaction catalyzed by Fe ions:

O_2_^●−^ + H_2_O_2_ → Fe^2+^/Fe^3+^ → ^●^OH + OH^−^ + O_2_

Enzymatic reactions, or the ones conducted in peroxisomes (e.g., β-oxidation), are the next important mechanisms generating ROS. In homeostasis, free radicals should be managed by components of antioxidant defense, like certain enzymes (e.g., glutathione). However, as a result of various agents (lack of antioxidant compounds in food, disease, and other environmental factors), the production of ROS may be higher than their elimination. An increase in the concentration of ROS leads to homeostasis disturbance and the formation of OS.

#### 3.1.2. Defense Mechanisms against ROS Overproduction

Through evolution, organisms have developed many ways to eliminate ROS and reduce inflammation. These are both exogenous and endogenous mechanisms.

Superoxide dismutase (SOD), catalase (CAT), glutathione peroxidase (GPx), glutathione S-transferase (GST), glutathione reductase (GSR), and thioredoxin reductase (TR) are enzymes balancing the generation of ROS in living organisms. They interact to scavenge ROS, and none of them can fulfill their function independently [28]. Superoxide dismutase and CAT serve as the first line of defense against OS, whereas TR, GPx, GSR, and GST constitute the second line [29,30]. Additionally, genetics play an important role in the battle against ROS. Nuclear factor erythroid 2-related factor 2 (Nrf2) has a central role in the regulation of antioxidant gene expression. It regulates the expression of more than 200 genes that encode proteins involved in antioxidant defense [29].

Exogenous substances that scavenge ROS are antioxidants from the diet. Antioxidant therapy was found to improve mitochondrial integrity. Additionally, substances like curcumin or vitamin C were linked to reducing OS [29,31].

Oxidative stress activates mechanisms of antioxidant defense [32]. In view of ROS overproduction and chronic inflammation, enzymes and compounds alleviating its effects may be insufficient. Therefore, every effort should be made to ensure that as few factors as possible enter the body to increase ROS production. That includes contaminants from the environment, e.g., MPs [33,34,35,36].

### 3.2. Consequences of Microplastics—Induced Oxidative Stress

The biochemical reactions that alter chemical constituents and reaction equilibria serve as the foundation for the theory explaining how MPs affect the development of OS. The phenomena seen at higher levels of biological complexity are explained by a cascade of branching changes that result in irreversible oxidative damage and the escalation of inflammatory processes [2]. According to research findings to date, MPs have direct toxic effects at the molecular, cellular, tissue, organ, individual, and population levels [17]. The toxicity of MPs is size-dependent [37], and the potential for ROS generation increases with plastic particle size [18,38]. Additionally, the generation of ROS rises when there are more MPs present.

#### 3.2.1. Formation of Oxidative Stress Due to the Effects of MPs

It has been shown that MPs can lead to the formation of ROS as a result of extracellular and intracellular processes [17,18]. Plastic degradation is connected to extracellular ROS [39]. Mechanical forces, temperature, light, chemicals, and biological variables contribute to the formation of MPs. These factors can act independently or in combination, leading to the formation of free radicals on the surfaces of plastics through various mechanisms [6,40]. For instance, this can involve the removal of a hydrogen atom from a macromolecular chain or the addition of groups of atoms to unsaturated bonds [41]. Secondary radicals (e.g., superoxide and alkyl radicals) are created when free radicals react with atmospheric oxygen, having an impact on living things.

Additionally, MPs (e.g., PS with 5 μm diameter, concentration of 50 μg/L) in intracellular processes result in ROS [42]. Through diffusion aided by transport proteins, passive transport across cell membranes, and endocytosis, plastic particles can be taken up by cells [43,44]. Microplastics are first transported to lysosomes and then, via the biofilm system, transported to the mitochondria, causing alterations in the potential of the mitochondrial membrane [45]. These contaminants are recognized as foreign substances and induce the defense of the innate immune system [42].

Oxidative stress is one of the most frequently reported negative effects that may result from exposure to MPs. Microplastics elicit OS in two different ways. First, MPs raise the level of ROS in body tissues, cells, and tissues. Additionally, as MPs alter the action of antioxidants including SOD, CAT, and glutathione (GSH), ROS cannot be efficiently removed, which also results in OS [46,47]. A change in antioxidant enzyme levels might be related to the size, type, concentration, and exposure time of MPs, as well as the trophic level of the tissues and organisms studied. Jeong et al. [48] observed the greatest effects for particles with the smallest diameter due to increased retention time and their higher bioavailability. *Paracyclopina nana* was treated with microbeads of PS at sizes of 0.5 and 6 μm and at concentration of 10 μg/L for 24 h. Fluorescent 0.5 μm PS-MPs were observed until 24 h post-ingestion, while 6 μm microbeads had disappeared. In contrast, Lu et al. [49] reported that larger MP particles (5 µm vs. 70 nm) induced a higher response after 7 days (20 mg/L). Wan et al. [50] investigated the changes induced by PS-MPs by analyzing antioxidant enzyme levels in larval zebrafish and noted an increase in MDA levels and a decrease in CAT, GSH, and total antioxidant capacity (T-AOC). Zebrafish were treated with 5 and 50 μm PS-MPs at concentrations of 100 and 1000 μg/L for 7 days. The content of GSH decreased significantly after exposure to 100 and 1000 μg/L MPs. In addition, the activity of CAT decreased significantly in larval zebrafish when exposed to 1000 μg/L PS-MPs. However, there was no significant change in the activity of SOD between the control group and both sizes of MPs in the treated groups. In contrast, Lu et al. [49] observed in their study on *Danio rerio* that SOD and CAT activity levels increased with rising concentrations of administered MP. Animals were treated with 5 μm PS-MPs and with exposure at concentrations of 20 μg/L, 200 μg/L, and 2000 μg/L for 7 days. Generally, the activities of SOD and CAT significantly increased in a dose-response manner. Thus, the dose size, which affected the subsequent effect, was another crucial factor.

#### 3.2.2. Causes of the Negative Impact of MPs on Organisms

Understanding the causes of various types of damage is necessary to comprehend how MPs affect living things. The activation of mitogen-activated protein kinase (MAPK) pathways is the underlying cause of MPs’ detrimental effects on health. Major cellular molecules like deoxyribonucleic acid (DNA) and lipids may malfunction or change in structure as a result of this. Increased lipid peroxidation (LPO), oxidized biomolecule formation, DNA mutations, and excess ROS all contribute to cell apoptosis [37]. Numerous diseases may result from these modifications.

The activation of MAPK pathways can be induced or mediated by ROS [51]. It was discovered that the size of the plastic particles affects how the MAPK pathway is activated. Findings after 24 h of exposure to PS microbeads of 0.5 and 6 μm at concentrations of 10 μg/mL in *Brachionus plicalitis* indicated that smaller particles activated more proteins that may mediate their toxic effects [18]. Gene transcription, protein synthesis, cell division, and cell apoptosis are just a few of the intracellular processes that are regulated by the family of enzymes known as mitogen-activated kinases. As a three-level cascade of enzymes that are successively activated as a result of phosphorylation, they take part in signaling pathways. A concurrent rise in Nrf2 activity, which controls the expression of antioxidant proteins that guard against oxidative damage, may regulate MAPK activation by ROS [52]. Crabs, namely *Eriocheir sinensis*, treated with 5 μm MPs at a concentration of 4000 μg/L [53], copepods, namely *Paracyclopina nana*, treated with 0.5 and 6 μm MPs at a concentration of 20 μg/mL [48], and Balb/c mice [47] treated with 5 μm MPs at a concentration of 1 mg/d exhibited activated MAPK pathways after being exposed to PS-MPs.

Microplastics are able to pass through a variety of biological barriers and come into contact with lipid membranes. It has been found that plastic particles adhere to lipid membranes. This causes the lipid bilayer to stretch significantly, which can seriously impair the functioning of the cellular apparatus [54]. Nevertheless, MPs can also result in oxidative processes that affect lipids, which can lead to LPO. This happens as a result of excessive ROS generation, which leads to the oxidation of polyunsaturated fatty acids (PUFA) by free radicals. This process results in damage to other lipid-containing structures, such as cell membranes. This process is linked to a number of pathologies and disease states, such as reactive aldehydes which are formed as a result of oxidation. They can combine with proteins and DNA to form adducts, which alter their function and result in a number of diseases, e.g., cancer, atherosclerosis, and neurodegenerative problems [55].

It was found that plastic particles affected LPO in the fish *Dicentrarchus labrax*, the coral *Coelogorgia palmosa*, and C57BL/6J mice [37,56,57]. In the case of the fish Dicentrarchus labrax, polymer microspheres (thermoset amino formaldehyde polymer, 1–5 μm at concentrations of 0.25 and 0.69 mg/L) were used, and increased LPO in the brain and muscles was observed [56]. In addition, exposure to MPs (PE, 180–212 µm, spheres, 50–70 mg/L) increased LPO in the coral *Coelogorgia palmosa*, indicating oxidative damage [57]. This effect was also observed when mice were treated with PS (0.5 and 5 μm, spheres, 10 mg/L) [37]. In these studies, all particles were spherical in shape. However, different polymers and particle sizes were used. This makes it difficult to compare the results obtained and demonstrates the need to standardize MPs studies.

Plastic particles can undermine the antioxidant defense mechanisms by increasing the production of ROS. Reactive oxygen species in turn cause DNA damage, which disrupts the genetic control of the proper DNA repair pathway [58,59]. Both the mitochondria and the nucleus can suffer DNA damage as a result of MP exposure [60].

Plastic particles induce DNA damage that is dependent on the particles’ size, concentration, and exposure time [61,62]. Çobanoğlu et al. (2021) treated human peripheral lymphocytes with different amounts of MPs (PE-MPs, 10–45 μm). They found that chronic stress in cells exposed to low concentrations of MPs (50 μg/mL) over a long period of time (48 h) resulted in genomic instability [63]. In an experiment conducted on human colon adenocarcinoma cells, Caco-2, an increase in ROS levels and cellular DNA damage were observed after incubation at various concentrations (20 and 200 μg/L) of 5 μm PS-MPs. Therefore, it was concluded that toxic effects were dependent on the MPs’ concentration [64].

Studies on DNA damage caused by MPs were conducted on both mussels, *Mytilus galloprovincialis* and *Scrobicularia plana* [65,66], and fish, *Oreochromis niloticus* [67]. In the first study, the organisms were treated with PS and PE particles, both less than 100 µm in size, at concentrations of 0.5, 5, and 50 μg/L [66]. In the second study, PS particles of 20 µm in size were used (100 mg/L) [65]. In the third study, there was no information about the type of MPs used, except that particles larger than 100 nm were employed and concentrations were 1, 10, and 100 mg/L [67]. We think that, when examining the effect of MPs on organisms, one of the fundamental requirements should be to provide the type of plastic. Depending on the type of particle, various effects of MPs on the tested cells or organisms can be identified. For instance, it has been found that PS caused an increase in the amount of ROS [68,69,70,71,72], while PET sizes 25 and 90 μm did not induce any changes in ROS generation [69].

According to the studies discussed so far, plastic particles can cause genotoxicity [73]. Age-related disorders may also be influenced by OS and DNA damage mediated by MPs [74]. Cancer and other genetic disorders can arise as a result of disruptions in the replication, transcription, and repair processes caused by DNA damage. The negative impact of MPs on DNA, however, is still the subject of limited scientific study. The mechanisms of DNA damage resulting from the interaction of MPs are not completely understood. Further research is needed on the MPs–DNA relationship. It is of critical importance to public health.

### 3.3. Research Approaches and Methodologies

The study of oxidative stress is realized via direct measurement of reactive oxygen species as well as evaluation of the reactions/effects caused by the factors that induce this condition. Techniques that can be classified under the categories of antioxidant status evaluation or oxidative damage assessment are employed to investigate the impacts of oxidative stress. The assessment of antioxidant status involves the measurement of antioxidant enzyme levels indicative of oxidative stress generation, whereas the research of oxidative damage encompasses, for instance, lipid peroxidation or DNA damage (including metabolic pathways). Section 3.3 presents the research methods used in the analysis of oxidative stress.

#### 3.3.1. Measurement of Reactive Oxygen Species

The effects of various xenobiotics on aquatic organisms can be assessed using OS indicators [75]. Reactive oxygen species are key molecules responsible for the damaging effects of OS. Measurements to estimate cellular ROS levels by using specific ROS/RNS fluorogenic probes have been used in the past [76,77]. Determination of H_2_O_2_, hydroxyl radicals (OH^•^), and superoxide radicals (ROO^•^) can be performed using 5(6)-carboxy-2′,7′-dichlorodihydrofluorescein diacetate (DCFDA). The membrane-permeable probe then readily diffuses across cell membranes and is hydrolyzed by intracellular esterases to the polar and non-fluorescent form of DCFH (2,7-dichlorodihydrofluorescein), which is retained intracellularly. The DCFH is then trapped in the cells and reacts with H_2_O_2_ to form fluorescent 2′,7′-dichlorofluorescein (DCF). The fluorescence intensity of DCF can be analyzed using flow cytometry or a fluorescence plate reader [78]. Superoxide (O_2_^−^) molecules, on the other hand, can be detected with another fluorescent probe, dihydroethidium (DHE) [79]. Hydroperoxides (R-OOH) are also determined by evaluating derivatives of reactive oxygen metabolites. The method uses the Fenton reaction, where hydroxyl groups are converted to alkoxy (RO^•^) and oxygen radicals (R-OO^•^). The resulting radicals are trapped in a chromogen (N, N -diethyl-para-phenylenediamine) by which the corresponding radical cation is formed. The concentration of newly formed radicals is directly proportional to the concentration of peroxides present in the test material, which is measured using spectrophotometric methods [80]. However, direct measurement of ROS levels can pose difficulties in achieving high accuracy and precision due to their short lifespan and rapid reactivity with components that regulate the redox state. This is related to the fact that, while ROO^•^ and H_2_O_2_ show relatively good stability (from a few seconds to a few minutes), it is OH^−^ that has a high reactivity (less than a nanosecond) [81].

**Figure 2:**
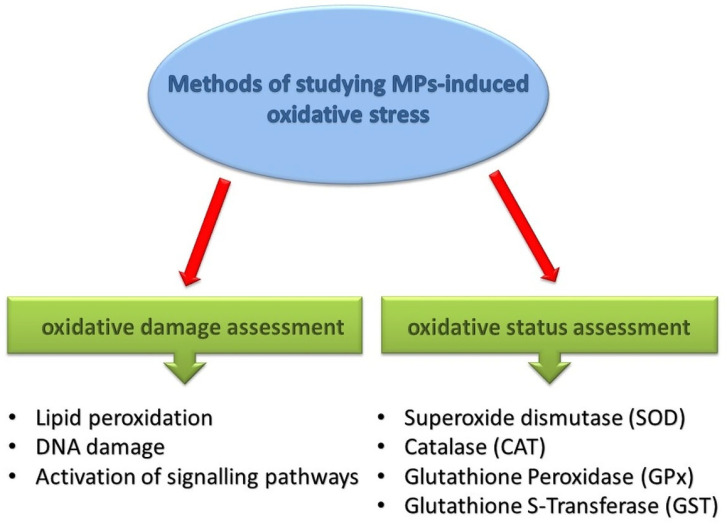
Scheme of methods for studying MPs-induced oxidative stress [2,81].

Therefore, indirect evaluation of ROS is carried out via oxidative damage assessment or antioxidant status assessment in order to evaluate MP-induced OS (Figure 2). To assess oxidative damage, studies on LPO, DNA damage, or activation of signaling pathways are used.

#### 3.3.2. Oxidative Damage Assessment–LPO Peroxidation

Overproduction of ROS causes excessive oxidation of cell membrane lipids. Malondialdehyde is one of the most well-studied end products of the peroxidation of PUFA and is often used in assessing OS conditions. It can be measured using the reagent thiobarbituric acid (TBARS) [82]. It reacts under reduced pH conditions at 100 °C to form a pink- or red-colored product, which is extracted with butanol and determined spectrophotometrically or fluorometrically. This method is quick and easy, but not specific, as other aldehydes (other than MDA) can react with TBARS and form derivatives that exhibit absorbance at the same wavelength [83]. Alternatively, MDA can be determined using high-performance liquid chromatography (HPLC) [84] and gas chromatography–mass spectrometry (GC-MS) [85], but it is more labor- and time-consuming.

Other LPO markers used are 8-iso-prostaglandin F2 α (8-iso-PGF2 α), 4-hydroxy-2-nonenal (4-HNE), conjugated diene (CD), and lipid hydroperoxides (LOOH), which provide various reliable approaches for their identification [81]. The 8-iso-PGF2 α, which is a product of non-enzymatic peroxidation of arachidonic acid in membrane phospholipids, can be determined using high-speed ultra-high performance liquid chromatography with tandem mass spectrometry (UHPLC-MS/MS). However, it is important to note the limitations of labor intensity and the need for specialized and expensive equipment [86]. In turn, the unsaturated hydroxyalkenal 4-HNE can be studied, preferably using immunohistochemistry (IHC) or HPLC [87,88]. Conjugated dienes formed as a result of the autoxidation of PUFA induced by free radicals can be determined spectrophotometrically [89]. LOOH, which constitutes the main PUFA oxidation product, can be determined using the xylenol iron oxidation (FOX) test. It is based on the ability of LOOH to oxidize ferrous iron in the presence of xylenol orange, leading to the formation of a colored iron–xylenol-orange complex, which is measured spectrophotometrically [90].

#### 3.3.3. Oxidative Damage Assessment—DNA Damage

The main types of DNA damage (or changes) caused by OS are base modifications, base sites (i.e., loss of base), and DNA strand breaks. The appropriate choice of test methods for measuring DNA damage varies depending on the subject of interest [91]. Currently, the base modification of 8-oxoguanine is being used to evaluate DNA oxidation products. Mass spectrometry (MS) or GC and LC with electrochemical detection (ECD) have been employed for this purpose [81]. The gold standard appears to be the combination of LC with tandem MS. However, despite their many advantages, these techniques also have drawbacks. A large amount of DNA is needed [92], as well as proper preparation of the DNA for testing, which causes artificial oxidative changes.

Another example of measuring oxidative base damage is Southern blot analysis. In this method, DNA is treated with restriction enzymes, followed by digestion with, e.g., glycosylase, placing it on an agarose gel, and measuring the stripe intensity with a gene-specific probe via Southern blot [93]. The advantage of this assay is the minimal processing required, which significantly reduces the number of artifacts produced [94]. On the other hand, a method that evaluates single-stranded and double-stranded DNA breaks (SSB or DSB, respectively) is measured using single-stranded gel electrophoresis (also known as the SCGE comet assay) [95]. Significant limitations of this assay are its sensitivity, reproducibility, and relatively low throughput [91]. Currently, the following methods are used to assess MP-induced DNA damage: electrophoresis [65], DNA microarray platform [66], or microplate reader [96].

On the other hand, to assess MPs-induced oxidative damage, it may be useful to study signaling pathways that can activate the body’s defense mechanism induced by OS. The study of the effect of microplastic particles on the induction of OS is realized by analyzing MAPK signaling pathways. The MAPK pathway activity is analyzed by determining the phosphorylation status of p38 kinase (p-p38) and c-Jun N-terminal kinase (p-JNK) after an appropriate incubation time [97]. The method used to assess the expression of antioxidant genes and those involved in the MAPK signaling pathway is qRT-PCR (Real-Time Quantitative Reverse Transcription PCR) [53].

Other examples of methods used to analyze MAPK signaling pathways applied to assess oxidative stress include ELISA, NFκB activation assay [98], phospho-flow cytometry [99], electrophoretic mobility shift assays (EMSA) [100], or Western Blot [101,102].

#### 3.3.4. Oxidative Status Assessment—Enzymatic Induction of Oxidative Stress

The main enzymes used in assessing antioxidant status are SOD, CAT, GPx, and GST. The first, SOD, is responsible for regulating ROS levels by catalyzing the conversion of superoxide to H_2_O_2_ and molecular oxygen [103]. Their total activity can be determined with a method based on the ability of SOD to inhibit the autoxidation of epinephrine to adrenochrome in an alkaline environment and is measured with spectrophotometry [104]. In another method, the ability of SOD to inhibit the autoxidation of pyrogallol to a yellow compound is tested and also measured spectrophotometrically [84]. In addition, there are also indirect methods to measure SOD activity. They rely on the fact that superoxide radicals, generated by the NADH/D-amino acid oxidase–phenazine–methosulfate (PMS) system or the xanthine–xanthine oxidase system, cause the reduction of tetrazolium salts to a blue compound, the absorbance of which is assessed spectrophotometrically [105].

The second enzyme, CAT, is responsible for the decomposition of H_2_O_2_ into water and oxygen [106]. One of the quantitative methods used is the spectrophotometric assay, which tracks the decomposition of H_2_O_2_ catalyzed by CAT by observing the decrease in ultraviolet absorption (of hydrogen peroxide) over time [107]. Another colorimetric method is based on the use of H_2_O_2_ by CAT using the reagent K_2_Cr_2_O_7_/acetic acid [108]. At elevated temperatures in the presence of hydrogen peroxide, dichromate in acetic acid is reduced to chromium acetate. The above methods determine CAT activity as a result of the disappearance of hydrogen peroxide. The unit of CAT is defined by the amount of 1 µmol of H_2_O_2_ decomposed per minute. Other spectrophotometric methods are based on the formation of a stable H_2_O_2_ complex with ammonium molybdate [109], or the peroxidation function of alcohol oxidation [110] is used.

Another antioxidant enzyme, GPx, catalyzes the reduction of H_2_O_2_ and lipid peroxides to water and lipid alcohols by oxidizing GSH to glutathione disulfide (GSSG) [108]. Ellman’s reagent, 5,5′-dithiobisnitrobenzoic acid (DTNB), is used to measure the amount of H_2_O_2_ consumed in order to determine the GSH and GPx levels [111]. In another method, GPx catalyzes the oxidation of glutathione by hydrogen peroxide, for example, where it is later reduced by exogenous glutathione reductase, causing the oxidation of the reaction coenzyme NADPH (the reduced form of nicotinamide adenine dinucleotide phosphate—NADP^+^) to NADPH^+^ [112].

The last enzyme belonging to the multigene isoenzyme family is GST. This isoenzyme has a catalytic role in coupling with various harmful electrophilic compounds for detoxification. In addition, many GST isoenzymes reduce LOOH and detoxify LPO end products (e.g., 4-HNE) [113]. The method for assessing GST activity is the ability of the isoenzyme to conjugate 1-chloro-2,4-dinitrobenzene (CDNB) to reduced glutathione. It is measured spectrophotometrically [112].

Numerous models have discussed the possibility that MPs molecules may be responsible for the increased ROS generation that is induced within cells [114]. These models ranged from fish cell lines to those of mammals, marine invertebrates, and live fish [42,115,116]. The kinds of cells or organisms used are crucial [45,68,71]. This effect was shown by Salimi et al. (2022) who noticed that PVC induced OS and organelle damage only in human lymphocytes. Fish lymphocytes did not experience any modifications. This demonstrated that human lymphocytes are more susceptible to the toxicity of MPs when compared to fish lymphocytes [117].

### 3.4. Effects of MP-Induced Oxidative Stress on Cells

**Figure 3:**
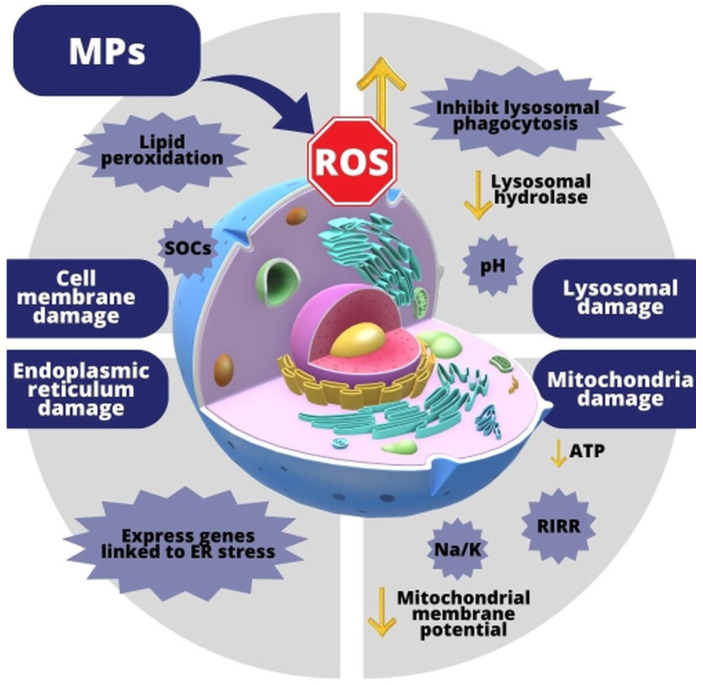
Effect of MPs on cells via OS (SOCs—the opening of store-operated Ca^2+^ channels; ATP—adenosine triphosphate; RIRR—ROS-induced ROS-release; Na/K—the opening of sodium/potassium transmembrane channels; and ER stress—endoplasmic reticulum stress).

Predicting the cytotoxicity of plastic particles requires an understanding of how they interact with cells (Table 1). Therefore, an examination of how MPs affect specific cellular organelles may be beneficial (Figure 3) [68,69,70,71,72,118,119].

#### 3.4.1. Effect of MPs on the Cell Membrane

The biological membrane that separates the interior of the cell from the outside environment is called the cell membrane. It serves as a barrier, obstructing substances’ free entry and exit into and out of the cell. Because of this, the intracellular environment is comparatively stable, enabling the orderly progression of biochemical reactions [125].

The cell membrane is the first defense against MPs being taken up by cells. Endocytosis, passive infiltration, phospholipid hydrolysis, or transport proteins found on the cell membrane can all be used to take up plastic particles [126,127].

The direct or indirect damage that MPs cause by raising the level of ROS is one of the mechanisms by which they have an effect on the cell membrane. When MPs come into contact with the cell membrane, they physically harm the cells. Hydrophobic interactions and Van der Waals forces allow for the adsorptive attachment of a significant number of particles to the membrane [126]. As a result, the total membrane surface area decreases, and its tension and thickness increase (PE and PMMA with a diameter of 8 to 10 μm, concentration of 500 μg/mL; PE, PS, and PMMA with a diameter of approx. 1 μm, concentration of 400–500 μg/mL) [54,128]. Additionally, particle size is important. It has been shown that PS particles in a 10 mg/mL solution with a size of 5 µm, in contrast to particles with a size of 0.5 µm, are unable to adhere to the cell membrane because of their size and weak Brownian motion [126]. Aggregates of MPs, however, can lead to pore formation [128]. Due to their role in the production of more ROS, MPs also harm the cell membrane’s structure. Even a brief exposure to MPs can result in an increase in intracellular ROS levels, according to in vitro research. The structure of the cell membrane is harmed as a result of the LPO that ROS cause (PVC with diameter from 0.16 to 1.82 μm, concentrations of 48 and 96 μg/mL) [117]. Furthermore, it has been demonstrated that PS with diameters of 3 and 10 µm (800 and 1600 MPs/mL) induced ROS in human hepatocytes undergoing apoptosis by opening Ca^2+^ channels on the cell membrane (store-operated Ca^2+^ channels‒SOCs) [123]. Cellular damage created by OS and apoptosis are frequent consequences of MPs (PP with a diameter of 8 μm and a concentration of 10 mg/mL) [129].

#### 3.4.2. Effect of MPs on Lysosomes

Cytoplasmic organelles, known as lysosomes, are encircled by a single lipid–protein membrane. They contain hydrolases whose primary function is the phagocytosis of aging cells or the digestion of foreign substances. These elements control the differentiation, division, and growth of cells. Age-related diseases, such as cancer and neurodegeneration, are caused by malfunctioning lysosome functions [130].

Reduced lysosomal hydrolase activity, a change in lysosomal pH, and dysfunctional autophagy are all signs of lysosome damage (4 μg/mL of 191.6 nm PET and 1.85 μm PET) [131]. These organelles may be harmed by MPs directly or indirectly.

Lysosomal damage can occur through direct means when cells ingest plastic particles (e.g., PS, 10 µg/mL) via endocytosis [132]. This ingestion can lead to attempts by the cell to digest the foreign particles, which, in turn, may result in lysosome disruption [132]. For examples, in vitro tests on model cell membranes and rat basophilic leukemia cells (RBL-2H3) have shown that PS (0.5 µm, 20 mg/L) accumulates in lysosomes [126].

The primary mechanism by which plastic particles are released from cells is exocytosis. Cell membrane disruption is an alternative to exocytosis [126]. Since lysosomal membranes are extremely vulnerable to the oxidative effects of ROS, the excessive production of ROS is what causes the indirect effect of MPs (e.g., 0.2 µm, 100 and 200 μg/mL) on lysosomes [133]. However, particle size is also crucial. Lysosomal membrane permeability in human intestinal cells HT-29 exposed to PS (3 µm, 800 MPs/mL) was observed under a microscope. Cells exposed to PS (10 µm, 800 MPs/mL) produced more ROS [123]. In addition, both common and blue mussels (*Mytilus galloprovincialis*—20 g/L PE, and PS, *Mytilus edulis*—50 µg PS/mL) exhibited lysosome dysfunction following contact with plastic particles [66,134]. Microplastics were taken up by the gills and the stomach, where they accumulated and caused histopathological changes as well as a significant inflammatory response, as evidenced by the destabilization of the lysosomal membrane [43]. The swollen lysosomes released cathepsins into the cytoplasm, which ultimately damaged the mitochondria and led to apoptosis (50 µg PS/mL) [135].

#### 3.4.3. Effect of MPs on Mitochondria

Eukaryotic, double-membraned mitochondria carry out vital cellular processes like respiration, energy production, and metabolism. The pathogenesis and development of numerous illnesses, such as cancer, stroke, ischemia, diabetes, obesity, heart disease, and neurodegenerative disorders, are influenced by mitochondrial damage [136,137].

The swelling of the mitochondria, weakening of the myelin substance in the mitochondrial membrane, reduction or disappearance of crista, decreased enzyme activity in the mitochondria, altered permeability of the mitochondrial membrane, and damage to the mitochondrial DNA are all morphological manifestations of MP damage [119,138,139].

Mitochondria are the primary sites of ROS production in cells, formed when ATP is made through the oxidative phosphorylation of carbohydrates and fatty acids [140,141]. The inner membrane (Complexes I–III), the matrix (dehydrogenases), and the outer membrane (monoamine oxidase) contain the primary ROS producers in mitochondria [140]. Damage to the mitochondria causes an overproduction of ROS and different oxidases. It has been shown that MPs (PS, 3.15–3.93 µm, 300 ng/mL) can induce cytotoxicity through OS by inhibiting heme oxygenase-1 (HMOX1), an antioxidant enzyme that is localized in this organelle [122]. Under OS, there is a loss of mitochondrial membrane integrity, the release of apoptotic factors, and the associated activation of caspase, which is an enzyme from the cysteine protease group that degrades cellular proteins by cutting peptide bonds (PS, 0.2 µm, 100 μg/mL) [124]. The permeability of the mitochondrial membrane is significantly changed by MPs (PVC; 0.16–1.82 μm; 24, 48, and 96 μg/mL) [117]. They alter the membrane’s structure by destroying the mitochondrial ETC, which lowers the membrane’s potential and causes depolarization of the mitochondria (PS, 0.5 µm, 100 µg/mL, 4 h of exposure) [121]. Excessive mitochondria exposure to ROS may also result in the opening of Na/K transmembrane channels, which disturbs the potential of the mitochondrial membrane [117]. Through the ROS-induced ROS-release (RIRR) mechanism, increased ion flux in membrane channels can further cause the collapse of the mitochondrial membrane potential and the release of ROS [140]. It is the process by which one cellular compartment or organelle produces or releases ROS, causing another compartment or organelle to produce or release more of it. The size of the plastic particles (PS, 0.117 µm, 1 mg/L) that cause damage to the mitochondrial membrane is related to that damage [97]. It has been established that MPs significantly inflict more membrane damage than NPs [121].

Multiple studies have demonstrated that plastic particles decrease the potential of the mitochondrial membrane [70,142]. Rotifers, namely *Brachionus koreanus*, a mouse spermatocyte line (GC-2 cells), and adult zebrafish (*Danio rerio*) all showed this effect [18,70,143]. Additionally, a study of the rotifer *Brachionus koreanus* indicated that PS (0.05 and 0.5 μm, 10 μg/mL) had an impact on the outer mitochondrial membrane by increasing the amount of ROS in cell compartments close to the mitochondria [18]. To investigate the impact of MPs on the mouse spermatocyte line, larger PS (5 µm, 25 mg/mL) particles were used. The findings demonstrated that the plastic particles reduced ATP production, compromised the mitochondrial genome’s integrity, and disturbed the balance between mitochondrial fusion and division. The autophagy pathway PINK1/Parkin in mitochondria was turned on [70]. In the liver of the zebrafish, exposure to MPs (<200 µm, 1 mg/L) also resulted in a deficit in mitochondrial respiration [143]. In contrast, PE (5–60 μm, 1 mg/mL) caused a reduction in cell viability and an increase in the response to OS, particularly the production of mitochondrial superoxides, in human colorectal adenocarcinoma cells Caco-2 and HT-29 [144]. Mitophagy and mitochondrial fission may be indirectly triggered by MPs’ induction (PS, 5 µm, 0.1 mg/day—about 1.46 × 10^6^ particles) of endoplasmic reticulum (ER) stress [145].

#### 3.4.4. Effect of MPs on an Endoplasmic Reticulum

The endoplasmic reticulum is a network of channels that is both intracellular and intercellular and is separated from the basal cytoplasm by biological membranes. It is under stress due to a variety of external factors, including MPs, which results in an increase in the amount of unfolded or misfolded proteins present there [146,147].

Polystyrene was the polymer that was most frequently used in studies on how MPs affected RS stress [145,148,149,150]. Rats, mice, and fish were used in these analyses [145,148,149,150]. It was discovered that PS (1 µm, 2.0 mg/kg) caused young rats to express genes linked to ER stress (PERK, eIF2α, ATF4, and CHOP) [150]. The stress of ER and oxidative damage were discovered to cause nephrotoxicity in other trials carried out on rats using the same kind of particle (PS, 1 µm, 2.0 mg/kg/d) [148]. Oral administration of PS (5 μm, 0.1 mg/day—about 1.46 × 10^6^ particles) to the mice strain C57BL/6 J resulted in OS, excessive ROS production, and liver ER stress. Therefore, MPs are hepatotoxic [145]. However, the results of the analyses conducted on carp showed that, in addition to causing ER stress, plastic particles also disrupt the intestinal microflora, cause inflammation in the intestinal tissue, and induce apoptosis [149].

### 3.5. Effects of MP-Induced Oxidative Stress on Tissues and Organs

Plastic particles have emerged as a brand-new class of environmental contaminants that can build up in a variety of tissues and organs [151], producing ROS and triggering OS (Table 2) [34,37,138,145,152,153,154,155,156,157]. For examples, damage to the liver and pancreas in *Lithopenaeus vannamei* resulted from an imbalance in the antioxidant system, and this damage worsened as the MP concentration increased (PS; 2 µm; 0.02, 0.2, and 1 mg/L) [157].

Tissue and organ defects were observed in various in vivo models following exposure to MPs [2,49,158]. The most prevalent were in the fish (e.g., PS; 5 µm; 20, 200, and 2000 µg/L) and mouse models (PS; 0.5 and 5 μm; 10 mg/L) [37,49].

Microplastics exposure led to physical damage to a fish’s digestive system and affected their respiratory system [82]. Microplastics can build up in a fish’s liver, intestines, and gills. Ingestion of plastic particles can result in inflammation in the liver and intestines [82]. However, compared to other organs, the liver is more vulnerable to oxidative conditions [159]. Plastic particles directly affected SOD and MDA in the gills, as well as SOD and CAT in the digestive system of the fish *Gambusia affinis* [82]. Yang et al. attempted to assess the effect of increasing exposure time to PS-MPs on the liver tissues of juvenile red crucian carp [160]. It has been found that the antioxidant enzyme activities of SOD, GST, and MDA initially increase and then decrease with increasing exposure time. Simultaneously, CAT activity showed a wave-like trend of increase and decrease. When tissues in the heart (PS, 500 µm, 5 and 50 mg/L) and kidneys (PVC and PE; 40–150 µm; 1, 10, and 100 mg/mL) were exposed to MPs, OS was also induced, leading to an antioxidant reaction [138,155]. Changed SOD, MDA, CAT, and GST activities suggested OS (PS; 5 µm; 20, 200, and 2000 µg/L) [49]. Similar effects were observed in a mouse model (*Mus musculus*) when PS with particle sizes of 5 and 20 μm induced hepatic lipid accumulation and inflammation (0.5 mg/day) [158]. Additionally, MPs significantly decreased the weight of the liver and the proportion of M1 macrophages compared to M2 macrophages in both models described (PS, 0.5 and 5 µm, 10 mg/L; PVC and PE, 40–150 µm, 1, 10, and 100 mg/mL) [37,155]. A high MPs concentration caused tissues to undergo apoptosis more frequently.

### 3.6. Effects of MP-Induced Oxidative Stress on Organisms

Microplastics are released into the environment and harm numerous organisms in different ways (Table 3). Marine organisms are especially vulnerable [34,56]. Plastic particles penetrate the organisms mainly through the gastrointestinal system [3]. The most frequent observations of MPs’ effects on organisms include stunted growth, reduced size (length and weight), negative effects on reproduction, developmental changes, and a shorter lifespan [161,162,163,164]. Oxidative stress brought on by plastic particles can result in the aforementioned effects.

#### 3.6.1. Inhibition of Growth and Reduction in Body Size

It has been shown that MPs cause growth rates to decrease, usually based on models of invertebrates [161,165]. A significant factor affecting the rate of MP-induced growth reduction is particle size. Studies on the rotifer *Brachionus koreanus* have shown that, in contrast to MPs of 6 μm, a marked decrease in growth rate was observed for particles of 0.5 μm at similar PS exposure concentrations (10 μg/mL) [18]. Comparable findings were reported for marine microalgae, *Skeletonema costatum*, in which 1 μm PVC particles significantly inhibited growth when compared to 1000 μm PVC (50 mg/L) [166].

In addition, the body length of nematodes (*Caenorhabditis elegans*) was found to decrease after treatment with PS (0.5, 1, 2, and 5 µm; 1 mg/L) [38]. Microplastics have been proven to increase the body’s production of ROS, which has negative effects like weight loss, in in vivo tests using rats (PS; 0.5 µm; 0.0.015, 0.15, and 1.5 mg/d) and the Chinese mitten crab *Eriocheir sinensis* (PS; 5 μm; 40 μg/L, 400 μg/L, 4000 μg/L and 40,000 μg/L) [46,53].

#### 3.6.2. Negative Effects on Reproduction and Developmental Changes

Excessive production of ROS and OS is associated with infertility [162,163]. It includes disturbed spermatogenesis, fertilization, folliculogenesis, and implantation [167,168]. The impact of MPs on reproduction was confirmed in aquatic organisms [169] and mammals [46,47,170,171,172]. Specific particles and their sizes, doses, and effects on reproduction are presented in Table 4. The higher the concentration of MPs in the environment, the greater the consequences for the reproductive system [173,174].

The main effect of MP exposure in males is sperm viability (decrease in number, diameter, and speed of sperm) [174]. Plastic particles present in organisms enter into the testicles and cause pathological histological changes. Microplastics are capable of bioaccumulation in the testis, altering spermatogenesis progression and inducing an inflammatory response [171]. In vitro research showed that plastic particles enter Sertoli and Leydig cells [171]. Plastic particles (PS-MPs) at a dose of 0.1 mg/d reduce semen quality via the NRF2-HO-1-NF_K_B pathway connected to OS [175]. In addition, they are linked to decreased testosterone levels caused by excessive production of ROS [47].

The activation of the NLRP3/caspase-1 pathway in response to OS in females is one of the causes of problems with reproduction induced by MPs. It is connected to pathological inflammation and excessive release of the cytokines interleukin-1β (IL-1β) and interleukin-18 (IL-xie18) [176]. Modification of the hypothalamic–pituitary–gonadal (HPG) axis is another feature of MPs exposure affecting the female reproductive system. It was found that *Oryzias melastigma*, when exposed to PS-MPs (10-μm) at a concentration of 2 μg/L, was characterized with decreased sex hormone activity (17β-estradiol and testosterone) and gametes development [177]. In addition, PS-MPs at a concentration of 100 μm/L increased OS in gonads [178].

Microplastics can cause reproductive toxicity by preventing the expression of genes involved in detoxification and reproduction as well as inducing OS [179]. Nematodes (*Caenorhabditis elegans*) after exposure to MPs at sizes of 1, 2, and 5 μm and a concentration of 1 mg/L, and green sea urchin embryos (*Lytechinus variegatus*) after exposure to PE granules randomly sampled by sieving the surface sand in Santos Bay, have both shown developmental changes [38,180]. It has been demonstrated that plastic particles promote abnormal embryonic development [180].

Microplastics are recognized as damaging the reproductive system and reproduction [171,181]. However, it is still unknown what shape and size of plastic particles are most susceptible to penetration into the reproductive system and produce the greatest toxic effects. Research and results concerning MPs and disturbances to reproductive systems are presented in Table 4. In these studies, mice and rats were fed MPs in amounts ranging from 0.0007 mg/day to 100 mg/day.

One of the problems associated with most papers on MPs is that plastic particles are used in in vitro and in vivo toxicology studies in too high doses. Such an overestimation of intake may stem from a paper published by Senathirajah et al. (2021) in which it was determined that people can consume up to 5 g of MPs/week [182]. However, the study was criticized because it contained errors leading to an overestimation of plastic particle intake [183]. In another study, Mohamed Nor et al. (2021) estimated the daily consumption of MPs at 0.000583 mg/person/day for adults. This intake can lead to an irreversible accumulation of MPs of up to 0.0407 µg/person for adults up to age 70 in body tissue for particle sizes of 1–10 µm [184]. Compared to these data, the studies cited in Table 1, Table 2, Table 3 and Table 4 use doses higher than the exposures that have been determined for humans according to current knowledge. Thus, the occurrence of analogous health consequences in humans is unlikely. However, it should be noted that current limitations in MPs detection methods make it difficult to fully estimate the environmental concentrations of this type of contaminant. In addition, the high concentrations of plastic particles used in these studies may reflect the combination of multiple exposure pathways in nature and simulate the expected increase in MPs contamination in the future. The opposite problem is that inadequately designed procedures for sample collection and processing may result in contamination, which could cause the quantity of MPs in samples to be overestimated. Sources of plastic contamination in typical laboratory procedures can include water (even Milli-Q water), consumables, airflow (e.g., exhaust), and dust [185]. The primary tool for determining the amount of contamination introduced during an experiment is blind testing, which must be performed and included in conclusions [186].

#### 3.6.3. Shorter Lifespan

Plastic particles can contribute to an increase in mortality in many groups of organisms. For example, the LC50 (lethal concentration for 50% of organisms) falls within the range of 41–52 µg PP/mL, depending on the developmental stage of Artemia salina [164].

Research is being conducted to link MPs to various disorders and diseases. Nevertheless, OS and an increased level of antioxidant enzymes are recurring results of MPs exposure [187,188,189]. Polystyrene-MPs are believed to have the most toxic ecological effect [190]. However, other MPs are not considered safe [191]. Future research should focus on the effects that different popular MPs, i.e., PE, PP, and PET containing aromatic rings (like PS), have on living things.

### 3.7. Perspectives

Exposure to microplastic particles in the diet has become a growing public health challenge due to the increasing problem of MPs’ accumulation in the environment. Also, MPs particles from food packaging materials are becoming a growing problem as they are directly consumed by humans [192,193].

Currently, information on adverse effects from available literature sources is very limited. In the case of cell-based assays, Caco-2 was most commonly studied [68,119,120]. A frequently occurring endpoint was the increase in ROS observed at MPs’ concentrations of 10–100 µg/mL [68,120]. However, an increase in ROS was also observed at 0.1 µg/mL [115]. These studies mainly focused on the use of PS in the form of microspheres with a diameter of 5–10 µm [115,120]. We believe that the concentrations of MPs used in studies of this type should be reduced to reflect actual exposure. In the future, we would suggest the need for studies on human erythrocytes with MPs in concentrations of about 1.6 µg/mL, since this is the amount of plastic particles found in human blood [9]. These studies would need to use particles in the form of fibers and fragments, since MPs of such shapes are most often found in the environment. Additionally, we think that future investigations on cells should utilize smaller particles with sizes close to the lower limit of the MPs definition (0.1–1 µm), as these particles, among others, are thought to be potentially hazardous to health because of their small dimensions and consequent capacity for migration. Moreover, other types of polymers, e.g., PA, PE, PET, and PP, would also need to be popularized in cell studies. In the case of other studies, mice or their tissues are commonly used as model organisms [37,129]. In these experiments, as in the case of cells, spherical PS with sizes usually in the range of 5–20 µm were most commonly used [37,158]. The most frequent result of these tests was an alteration in the activity of antioxidant enzymes. We believe that future research should focus on tissues/organs of the gastrointestinal tract, as they are particularly affected by MPs due to widespread food contamination. Similar to cell studies, it would be suitable to employ fibers and fragments, reduce particle sizes, modify polymers, and lower concentrations in studies of tissues or organs to mimic actual exposures.

Studies on the toxicity of MPs conducted to date have shown that MPs exposure caused intestinal damage, liver infection, flora imbalance, lipid accumulation, and subsequently led to metabolic disorders [194]. Furthermore, MPs exposure increased the expression of inflammatory factors, inhibited acetylcholinesterase activity, reduced germ cell quality, and affected embryonic development [15,194]. Thus, we speculate that exposure to MPs may be involved in the development of various chronic diseases.

Although scientists have discovered some toxic effects of MPs exposure using a number of experimental models, they do not always reflect the actual concentrations. Thus, it would be appropriate to assess the influence of MPs more realistically from the point of view of their environmental concentration and the entire life cycle of organisms. There is a need for further research to understand the long-term effects of MPs. Future toxicity studies based on different doses of MPs and longer exposure times are recommended.

Due to the high surface energy of MPs, they also adsorb other pollutants, especially heavy metals (mercury, aluminum, iron, or zinc) and organic compounds (phenanthrene, benzo(a)pyrene, and others) [34]. Therefore, the mechanisms and environmental significance of the interaction between MPs and other pollutants are likely to become an important area of future research [10,34].

The issue of MPs toxicity requires further research to see if there is a synergy between plastic microbeads and adsorbed pollutants and the toxic mechanism. Currently, studies on MPs’ toxicity are mainly based on effects analysis, and the molecular mechanism is not fully elucidated. In addition, almost all MPs toxicity studies use experimental models and the harm to the human body is still unclear. Therefore, it becomes valuable to collect epidemiological and clinical data. Biomarkers can be used to investigate the intrinsic relationship between exposure to MPs and possible adverse health effects.

There should be a constant effort to pursue environmental sustainability by dealing with environmental problems caused by MPs, technology, product recycling, or upgrading the industrial structure. In addition, efforts should be made to strengthen national and world legislation and improve industrial standards, which constitute an indispensable part of controlling MPs pollution. Since plastic pollution is closely related to human activities, everyone should take care on their own to reduce the accidental dumping of plastic products and to dispose of them properly for the safety of the environment and human health.

## 4. Conclusions

The foundation for the toxic effects that MPs have on most organisms is the induction of OS. Lipid peroxidation, DNA damage, destruction of the morphology and function of mitochondria, an increase in cellular inflammation, and apoptosis can all be results of increased ROS production. The aforementioned damage will be made worse by the higher ROS generation that develops as a result of the lower mitochondrial membrane potential and mitochondrial depolarization caused by ROS. Thus, MPs have an impact on cells, tissues, organs, and whole organisms. Nevertheless, there is currently no proof of MPs’ direct impact on human health. It is essential to comprehend, among other things, the interaction between MPs and OS in order to evaluate the full range of risks related to plastic particles.

This comprehensive evaluation exposed notable variability in the reported connection between MPs and OS. It probably indicates that alternative redox-independent triggering mechanisms also have a significant role in the observed effects induced by plastic particles. In addition, this inconsistency may stem from the absence of standardized analytical criteria for comparing the obtained test results. The lack of a standardized analytical methodology, the use of various research models, and the wide variety of MPs (polymer type, size, shape, and surface charge) may be to blame for the variable and occasionally contradictory risk assessment findings from various studies. These factors are essential for determining how plastic particles affect ecosystems and human health. Additionally, in many scientific papers concerning MPs, a common issue is the utilization of excessively high doses of plastic particles in in vitro and in vivo studies. Consequently, the likelihood of analogous effects occurring in humans to those observed in cells or animals is low. However, it is important to note that current limitations in MPs’ detection methods hinder the full estimation of environmental levels of this contaminant. Moreover, the elevated concentrations of plastic particles employed in these studies may mirror the amalgamation of multiple exposure pathways in nature and simulate the anticipated increase in MPs contamination in the future.

For the purpose of creating a standardized research methodology with a public health focus, more research is required, particularly on mammal models. It would be beneficial to concentrate on how harmful MPs are when combined with other chemical pollutants and, ultimately, on ways to lessen the environmental damage caused by MPs and related OS. These topics are particularly relevant because MPs food contamination is increasingly being found.

## Figures and Tables

**Table 1:** Effects of MPs on cells via OS.

Cells	MP Type	MP Size	MP Shape	Dose	Exposure Time	Association (OS vs. Endpoints)	Reference
Human cells							
A549 cells	PTFE	6 and 31.7 μm	Fragments	10, 100, 500, and 1000 μg/mL	24 h	ROS increase (6 µm, 10 μg/mL); ROS decrease (31.7 µm, 1000 μg/mL); Increase secretion of IL-6 (6 µm, 1000 μg/mL; 31.7 µm, 10, 1000 μg/mL)	[68]
Caco-2 cells	PS	0.3, 0.5, 1, 3, and 6 μm	Spheres	20, 50, 70, 90, and 120 μg/mL	24 h	ROS increase (20, 50, 70, 90, and 120 μg/L); Increased mitochondrial membrane potential (20, 50, 70, 90, and 120 μg/L); Cytotoxicity (0.3 µm, 20, 50, 70, 90, 120 μg/mL; 0.5 µm, 120 µg/mL, 1µm, 90, 120 µg/mL; 3 µm, 70, 90, 120 µg/mL; 6 µm, 50, 70, 90, and 120 μg/mL)	[71]
Caco-2 cells	PS	0.1 and 5 µm	Spheres	1, 10, 40, 80, and 200 µg/mL	12 h	ROS increase (200 µg/mL)	[120]
Caco-2 cells	PS	0.2 and 2 μm	Spheres	10, and 100 µg/mL	24 h	Decrease in intracellular H_2_O_2_ levels (0.2 µm, 10 µg/mL); Differential expressions of redox-related genes, including HMOX1, CAT, and GPx1 (2 µm, 100 µg/mL)	[119]
Caco-2 cells	PS	8.9 µm and 1.14 µm	Fibers/ Fragments	10 and 100 µg/mL	24 h	Decrease in intracellular H_2_O_2_ levels (10 and 100 µg/mL)	[119]
Caco-2 cells	PTFE	6 and 31.7 μm	Fragments	10, 100, 500, and 1000 μg/mL	24 h	ROS increase (6 µm; 100 μg/mL); ROS decrease (31.7 µm, 1000 μg/mL); Nitric oxide induction (6 µm, 500 μg/mL; 31.7 µm, 1000 μg/mL)	[68]
CCD841CoN	PS	0.1, 0.5, 1, and 5 µm	Spheres	12.5, 25, 50, and 100 μg/mL	1 h	ROS increase (0.5 µm, 100 μg/mL)	[121]
CCD841CoN	PS	0.1, 0.5, 1, and 5 µm	Spheres	12.5, 25, 50, and 100 μg/mL	0.5 h	ROS decrease (0.1 µm, 50 and 100 μg/mL; 0.5 µm, 25, 50, and 100 μg/mL; 5 µm, 50 and 100 μg/mL)	[121]
HaCaT cells	PTFE	6 and 31.7 μm	Fragments	10, 100, 500, and 1000 μg/mL	24 h	ROS decrease (6 µm, 500 and 1000 μg/mL; 31.7 µm, 1000 μg/mL); Nitric oxide induction (6 µm, 10, 100, and 1000 μg/mL; 31.7 µm, 10 μg/mL)	[68]
HeLa cells	PE	3–16 µm	Spheres	from 0.01 to 10 μg/mL	24 h	Cytotoxicity (0.05, 0.1, 1, and 10 μg/mL)	[115]
HeLa cells	PS	10 µm	Spheres	from 0.01 to 10 μg/mL	24 h	ROS increase (10 μg/mL); Cytotoxicity (0.05, 0.1, 1, and 10 μg/mL)	[115]
HEK293 cells	PS	3.15–3.93 µm	Spheres	300 ng/mL	-	Decreased activity of HMOX1 (300 ng/mL); Cytotoxicity (300 ng/mL)	[122]
HIEC-6	PS	0.1, 0.5, 1, and 5 µm	Spheres	12.5, 25, 50, and 100 μg/mL	4 h	ROS increase (0.1 µm, 100 μg/mL; 0.5 µm, 100 μg/mL; 5 µm, 25, 50, and 100 μg/mL)	[121]
HIEC-6	PS	0.1, 0.5, 1, and 5 µm	Spheres	12.5, 25, 50, and 100 μg/mL	8 h	ROS increase (0.1 µm, 100 μg/mL; 0.5 µm, 25, 50, and 100 μg/mL; 5 µm, 100 μg/mL)	[121]
HIEC-6	PS	0.1, 0.5, 1, and 5 µm	Spheres	12.5, 25, 50, and 100 μg/mL	24 h	ROS increase (0.1 µm, 50 and 100 μg/mL)	[121]
HT-29 cells	PS	3 and 10 µm	Spheres	800 and 1600 MPs/mL	7 d	ROS decrease (3 µm, 1600 MPs/mL; 10 µm, 1600 MPs/mL); ROS increase (3 µm, 800 MPs/mL; 10 µm, 800 MPs/mL)	[123]
HT-29 cells	PS	3 and 10 µm	Spheres	800 and 1600 MPs/mL	14 and 21 d	ROS decrease (800 and 1600 MPs/mL)	[123]
HT-29 cells	PS	3 and 10 µm	Spheres	800 and 1600 MPs/mL	28 d	ROS decrease (3 µm, 1600 MPs/mL) ROS increase (3 µm, 800 MPs/mL; 10 µm, 800 MPs/mL; 10 µm, 1600 MPs/mL)	[123]
HT-29 cells	PS	3 and 10 µm	Spheres	800 and 1600 MPs/mL	48 d	ROS increase (3 µm, 800 and 1600 MPs/mL; 10 µm, 800 and 1600 MPs/mL	[123]
Human lymphocytes	PVC	0.16–1.82 μm	Spheres	24, 48, and 96 μg/mL	1 h	ROS increase (48 and 96 μg/mL); Increased activity of GSSG (24, 48, and 96 μg/mL); Decreased activity of GSH (24, 48, and 96 μg/mL); Mitochondrial membrane potential collapse (24, 48, and 96 μg/mL)	[117]
Human lymphocytes	PVC	0.16–1.82 μm	Spheres	24, 48, and 96 μg/mL	2 and 3 h	ROS increase (24, 48, and 96 μg/mL); Increased activity of GSSG (24, 48, and 96 μg/mL); Decreased activity of GSH (24, 48, and 96 μg/mL); Mitochondrial membrane potential collapse (24, 48, and 96 μg/mL)	[117]
Human lymphocytes	PVC	0.16–1.82 μm	Spheres	12, 25, 50, and 100 μg/mL	3 h	Cytotoxicity (25, 50, and 100 μg/mL)	[117]
Human macrophage	PS	0.2 µm	Spheres	100 µg/mL	24 h	The accumulation of lipids droplets in the cytoplasm (100 μg/mL)	[124]
Human-originated cardiac organoids	PS	1 µm	Spheres	0.025, 0.25, and 2.5 µg/mL	24 h	Decrease in ATP content (0.025, 0.25, and 2.5 µg/mL); SOD reduction (0.025, 0.25, and 2.5 µg/mL); Cytotoxicity (0.25 and 2.5 µg/mL)	[72]
Jurkat cells	PTFE	6 and 31.7 μm	Fragments	10, 100, 500, and 1000 μg/mL	48 h	ROS increase (6 µm, 10 μg/mL; 31.7 µm, 10 and 100 μg/mL); Nitric oxide induction (31.7 µm, 10 and 1000 μg/mL)	[68]
THP-1 cells	PTFE	6 and 31.7 μm	Fragments	10, 100, 500, and 1000 μg/mL	48 h	ROS increase (6 µm, 10, 100, 500, and 1000 μg/mL; 31.7 µm, 10 and 500 μg/mL); Nitric oxide induction (6 µm, 500 and 1000 μg/mL)	[68]
THP-1 cells	PS	0.5–1 μm and 8–10 μm	Fragments	62.5, 125, and 250 μg/mL	24 h and 72 h	Activation of NLRP3 inflammasome (250 μg/mL); Increased levels of IL-1β and MIP-1β (62.5, 125, and 250 μg/mL)	[5]
THP-1 cells	PS	0.5 μm and 3 μm	Spheres	62.5, 125, and 250 μg/mL	24 h and 72 h	Activation of NLRP3 inflammasome (250 μg/mL) Increased levels of IL-1β and MIP-1β (62.5, 125, and 250 μg/mL)	[5]
T98G cells	PE	3–16 µm	Spheres	from 0.01 to 10 μg/mL	24 h	ROS increase (0.05, 0.1 μg/mL); Cytotoxicity (0.05, 0.1, 1, and 10 μg/mL)	[115]
T98G cells	PS	10 µm	Spheres	from 0.01 to 10 μg/mL	24 h	ROS increase (0.05, 0.1, 1, and 10 μg/mL); Cytotoxicity (0.05, 0.1, 1, and 10 μg/mL)	[115]
U937 cells	PTFE	6 and 31.7 μm	Fragments	10, 100, 500, and 1000 μg/mL	48 h	ROS increase (6 µm, 100, 500, and 1000 μg/mL)	[68]
Animal cells							
GC-2 cells	PS	5 µm	Spheres	25 mg/mL	6 h	ROS increase (25 mg/mL); Decrease in ATP content (25 mg/mL)	[70]
GC-2 cells	PS	5 µm	Spheres	25 mg/mL	12 h	ROS increase (25 mg/mL)	[70]
GC-2 cells	PS	5 µm	Spheres	25 mg/mL	18 h	ROS increase (25 mg/mL)	[70]
GC-2 cells	PS	5 µm	Spheres	25 mg/mL	24 h	ROS increase (25 mg/mL); Reduction in mitochondrial membrane potential (25 mg/mL); Activation of the mitochondrial autophagy pathway PINK1/Parkin (25 mg/mL)	[70]
RTG-2 cells	PVC	25 and 90 μm	Spheres	1 mg/mL	24 h	ROS increase (1 mg/mL)	[69]
RTgill-W1 cells	PVC	25 and 90 μm	Spheres	1 mg/mL	24 h	ROS increase (1 mg/mL)	[69]
RTL-W1 cells	PVC	25 and 90 μm	Spheres	1 mg/mL	24 h	ROS increase (1 mg/mL)	[69]
Skin cells mouse (fibroblasts, keratinocyte)	PS	0.2, 1, 2 and 6 µm	Spheres	100 µg/mL	24 h	ROS increase (100 µg/mL)	[74]

A549—human lung adenocarcinoma; Caco-2—human colorectal adenocarcinoma cell; CCD841CoN—cells isolated from normal human colon tissue; HaCaT—human keratinocyte; HeLa cells—immortalized cell line; HEK293 cells—immortalized human embryonic kidney cells; HIEC-6—human intestinal epithelial cell-6; HT-29—human colorectal adenocarcinoma cell; Jurkat cell—an immortalized line of human T lymphocyte cells; THP-1—a human monocytic cell line derived from an acute monocytic leukemia patient; T98G cells—a glioblastoma cell line; U937—a cell line exhibiting monocyte morphology; B cells—secondary antigen-presenting cells, typically known to secrete antibodies; NK cells—natural killer cells; GC-2 cells—a mouse spermatocyte line; RTG-2 cells—the rainbow trout ovary cell line; RTgill-W1—the rainbow trout gill cell line; RTL-W1—the rainbow trout liver cell line; PS—polystyrene; PTFE—polytetrafluoroethylene; PE—polyethylene; PVC—poly(vinyl chloride); ROS—reactive oxygen species; MAPK—mitogen-activated protein kinase; IL-6—interleukin-6; CAT—catalase; HMOX1—Heme oxygenase 1; GPx1—Glutathione peroxidase 1; GSSG—oxidized glutathione; ATP—adenosine triphosphate; GSH—glutathione; NLRP3—the pyrin domain-containing protein 3; IL-1β—interleukin-1β; MIP-1β—macrophage inflammatory protein-1 beta; IFN-γ—interferon-gamma; TNF-α—tumor necrosis factor-α; IL-33—interleukin 33; IL-4—interleukin 4; IL-5—interleukin 5; IL-10—interleukin 10; IL-18—interleukin 18; TGF-β1—transforming growth factor β1; h—hour; d—day.

**Table 2:** Effects of MPs on tissues and organs via OS.

Tissues/Organs	MP Type	MP Size	MP Shape	Dose	Exposure Time	Association (OS vs. Endpoints)	Reference
Mice							
Mice intestinal tract	PP	8 and 10 µm	Fragments	0.1, 1.0, and 10 mg/mL	28 d	Increased activity of MDA (0.1, 1.0, and 10 mg/mL) and GSSG (1.0, and 10 mg/mL); Decreased activity of CAT, SOD, GSH, and GPx (0.1, 1.0, and 10 mg/mL); Activation of the TLR4/NFκB inflammatory signal pathway (0.1, 1.0, and 10 mg/mL)	[129]
Mice liver tissue	PS	0.5 and 5 μm	Spheres	10 mg/L	3 m	Decreased activity of SOD, GPx, and CAT (10 mg/L); Reduction in the expression of proteins related to oxidative stress, SIRT3, and SOD2 (10 mg/L)	[37]
Mice liver tissue	PS	5 µm	Spheres	0.01 mg/day (1 × 10^5^ MPs)	28 d	Increased activity of CAT, GPx, and AChE (0.01 mg/day)	[158]
Mice liver tissue	PS	5 µm	Spheres	0.1 mg/day (1 × 10^6^ MPs) and 0.5 mg/day (5 × 10^6^ MPs)	28 d	Decreased activity of CAT (0.1 mg/day and 0.5 mg/day); Increased activity of SOD, GPx, and AChE (0.1 mg/day and 0.5 mg/day)	[158]
Mice liver tissue	PS	20 µm	Spheres	0.01 mg/day (2 × 10^3^ MPs)	28 d	Increased activity of SOD, GPx, and AChE (0.01 mg/day)	[158]
Mice liver tissue	PS	20 µm	Spheres	0.1 mg/day (2 × 10^4^ MPs) and 0.5 mg/day (1 × 10^5^ MPs)	28 d	Decreased activity of CAT (0.1 mg/day and 0.5 mg/day); Increased activity of SOD, GPx, and AChE (0.1 mg/day and 0.5 mg/day)	[158]
Mice lung tissue	PS	5 μm	Spheres	1.25 and 6.25 mg/kg	3 times a week for 3 w	Decreased activity of SOD (6.25 mg/kg) and GPx (1.25 and 6.25 mg/kg)	[156]
Mice myocardial tissue	PS	500 μm	Spheres	0.5, 5, and 50 mg/L	90 d	Increased levels of MDA (5 and 50 mg/L); Decreased activity of SOD, GPx, and CAT (5 and 50 mg/L); Morphological changes in mitochondria (5 and 50 mg/L)	[138]
Other							
Clam digestive gland (*Scrobicularia plana*)	PS	20 µm	Spheres	1 mg/L	3 d	Increased activity of GPx (1 mg/L)	[65]
Clam digestive gland (*Scrobicularia plana*)	PS	20 µm	Spheres	1 mg/L	14 d	Increased activity of SOD (1 mg/L)	[65]
Clam digestive gland (*Scrobicularia plana*)	PS	20 µm	Spheres	1 mg/L	21 d	Increased activity of SOD (1 mg/L); Decreased activity of CAT, GPx, and GST (1 mg/L)	[65]
Clam gills (*Scrobicularia plana*)	PS	20 µm	Spheres	1 mg/L	3 d	Increased activity of CAT and GPx (1 mg/L)	[65]
Clam gills (*Scrobicularia plana*)	PS	20 µm	Spheres	1 mg/L	7 d	Increased activity of SOD (1 mg/L)	[65]
Clam gills (*Scrobicularia plana*)	PS	20 µm	Spheres	1 mg/L	14 d	Increased activity of SOD and GST (1 mg/L)	[65]
Clam gills (*Scrobicularia plana*)	PS	20 µm	Spheres	1 mg/L	21 d	Increased activity of SOD and GPx (1 mg/L)	[65]
Crab liver tissue (*Eriocheir sinensis*)	PS	0.5 µm	Spheres	40 and 400 μg/L	7 d	Increased activity of SOD, GSH, GPx, and GOT (40 and 400 μg/L); Decreased activity of CAT, AChE, GPT, GST, and MDA (40 and 400 μg/L)	[53]
Crab liver tissue (*Eriocheir sinensis*)	PS	0.5 µm	Spheres	4000 and 40,000 μg/L	7 d	Increased activity of MDA (4000 and 40,000 μg/L); Decreased activity of CAT, SOD, AChE, GOT, GPT, GPx, GSH, and GST (4000 and 40,000 μg/L)	[53]
Shrimp (*Litopenaeus vannamei)* liver tissues	PS	2 μm	Spheres	0.02 mg/L	8 d	Increased levels of SOD and GPx (0.02 mg/L); Decreased activity of CAT (0.02 mg/L)	[157]
Shrimp (*Litopenaeus vannamei*) pancreas tissues	PS	2 μm	Spheres	0.2 and 1 mg/L	8 d	Increased levels of MDA, SOD, and GPx (0.2 and 1 mg/L); Decreased activity of CAT (0.2 and 1 mg/L)	[157]
Zebrafish brain (*Danio rerio*)	EP	<200 µm	Fragments	0.1 and 1 mg/L	21 d	Increased activity of CAT, GSH, and GSSG (1 mg/L); Decreased of LDH (1 mg/L)	[143]
Zebrafish gut (*Danio rerio*)	PS	5 µm	Spheres	50 μg/L and 500 μg/L	21 d	Increased activity of CAT, SOD, and D-lactate (50 μg/L and 500 μg/L); Decreased activity of DAO (50 μg/L and 500 μg/L)	[42]
Zebrafish liver (*Danio rerio*)	PS	5 µm	Spheres	20 µg/L (2.9 × 10^2^ particles/mL)	7 d	Increased activity of CAT (20 µg/L)	[49]
Zebrafish liver (*Danio rerio*)	PS	5 µm	Spheres	200 µg/L (2.9 × 10^3^ particles/mL) and 2000 µg/L (2.9 × 10^4^ particles/mL)	7 d	Increased activity of CAT and SOD (200 µg/L and 2000 µg/L)	[49]
Zebrafish liver (*Danio rerio*)	EP	<200 µm	Fragments	0.1 and 1 mg/L	21 d	Increased activity of CAT and SOD (1 mg/L); Decreased activity of GPx and GST (1 mg/L); Decreased mitochondrial membrane potential (1 mg/L)	[143]

PS—polystyrene; PE—polyethylene; EP—ethylene–propylene copolymer; MDA—malondialdehyde; GSSG—oxidized glutathione; CAT—catalase; SOD—superoxide dismutase; GSH—glutathione; GPx—glutathione peroxidase; TLR4—toll-like receptor 4; NFκB—nuclear factor kappa B; ROS—reactive oxygen species; SIRT3—sirtuin 3; SOD2—superoxide dismutase 2; AChE—acetylcholinesterase; ER—endoplasmic reticulum; GST—glutathione S-transferase; GOT—aspartate transaminase; GPT—alanine aminotransferase; LDH—lactate dehydrogenase; DAO—diamine oxidase; d—day; NI—not indicated.

**Table 3:** An overview of studies where the biological effects of MPs on organisms via OS have been examined.

Organism	MP Type	MP Size	MP Shape	Dose	Exposure Time	Association (OS vs. Endpoints)	Reference
Benthic mollusc (*Pomacea paludosa)*	PP	11.86–44.62 μm	Spheres	250, 500, and 750 mg/kg	28 d	ROS increase (250, 500, and 750 mg/kg); Lipid peroxidation (250, 500, and 750 mg/kg); Impairs the biochemical parameters of CAT and GPx (250, 500, and 750 mg/kg); Reduced GSH and GST (250, 500, and 750 mg/kg)	[1]
*Caenorhabditis elegans*	PS	0.5, 1, 2, and 5 µm	Spheres	1 mg/L	3 d	Increase in gst-4p: GFP expression (1 mg/L)	[2]
Coral (*Coelogorgia palmosa*)	PE	180–212 µm	Spheres	50–70 mg/L	2 d	Increased activity of CAT, SOD, and GSR (50–70 mg/L); Lipid peroxidation (50–70 mg/L)	[3]
*Daphnia magna*	carboxylate-modified PS	0.3 µm	Spheres	1 mg/L	2 d	Increased activity of SOD (1 mg/L); Decreased activity of GSH (1 mg/L); Increased levels of MDA (1 mg/L); Reduction in AChE (1 mg/L)	[4]
Larval zebrafish (*Danio rerio*)	PS	5 and 50 µm	Spheres	100 and 1000 µg/L	7 d	Decreased activity of GSH (100 and 1000 µg/L); Decreased activity of CAT (1000 µg/L)	[5]
Marine copepod (*Paracyclopina nana*)	PS	0.5 µm	Spheres	20 mg/mL	1 d	Increased activity of GSR, SOD, GST, and GPx (20 mg/mL)	[6]
Marine copepod (*Paracyclopina nana*)	PS	6 µm	Spheres	20 mg/mL	1 d	Increased activity of SOD, GST, and GPx (20 mg/mL)	[6]
Marine copepod (*Tigriopus japonicus)*	PS	2 µm	Spheres	0.5 μg/L and 100 mg/L	30 d	ROS increase (0.5 μg/L and 100 mg/L)	[7]
Marine microcrustacean *(Artemia salina)*	PS	11.86–44.62 μm	Spheres	1, 25, 50, 75, and 100 μg/mL	2 d	Increased activity of SOD, CAT, GST, and GSH (1, 25, 50, 75, and 100 μg/mL); Reduction in AChE activity (1, 25, 50, 75, and 100 μg/mL)	[8]
Monogonont rotifer (*Brachionus koreanus*)	PS	0.5 μm	Spheres	10 μg/mL	1 d	ROS increase (10 μg/mL); Increased activity of SOD, GSR, and GST (10 μg/mL); Decreased activity of GSH (10 μg/mL)	[9]
Monogonont rotifer (*Brachionus koreanus*)	PS	6 μm	Spheres	10 μg/mL	1 d	ROS increase (10 μg/mL); Increased activity of GST (10 μg/mL); Decreased activity of GSH and SOD (10 μg/mL)	[9]
Nematode (Caenorhabditis elegans)	PS	1 μm	Spheres	1 mg/L	3 d	Induced oxidative stress (1 mg/L); Enhanced the expression of GST-4 (1 mg/L)	[2]

PS—polystyrene; PE—polyethylene; PP—polypropylene; ROS—reactive oxygen species; CAT—catalase; SOD—superoxide dismutase; GPx—glutathione peroxidase; GSH—glutathione; GST—glutathione S-transferase; gst-4—gene which encodes Glutathione S-transferase 4 (GST-4); GFP—green fluorescence protein; SOD—superoxide dismutase; GSR—glutathione reductase; GOT—aspartate transaminase; GPT—alanine aminotransferase; MDA—malondialdehyde; AChE—acetylcholinesterase; d—day; NI—not indicated.

**Table 4:** Effects of MPs on reproduction via OS.

Sex	Organism	MPs Type	MPs Size	MPs Shape	Dose	Exposure Time	Association (OS vs. Endpoints)	Reference
Mammals–Female	Rats	PS	0.5 μm	Spheres	1, 5, and 25 μg/mL (0.015, 0.15, and 1.5 mg/d)	90 d	Increased levels of MDA (0.015, 0.15, and 1.5 mg/d); Decreased the level of SOD (0.15 and 1.5 mg/d), GPx, and CAT (0.015, 0.15, and 1.5 mg/d); Fibrosis and granulosa cells apoptosis of ovary (5 and 25 μg/mL)	[1]
	Mice	PS	0.8 μm	Spheres	30 mg/kg/d	35 d	Increased level of ROS in oocytes (30 mg/kg/d); Reduced level of MDA (30 mg/kg/d); Increased IL-6 concentration in ovaries (30 mg/kg/d); Decreased viability of oocytes (30 mg/kg/d); Induced inflammation of ovaries (30 mg/kg/d)	[2]
Mammals–Male	Mice	PS	5 μm	Spheres	0.1, 1, and 10 μg/mL (0.7, 7, and 70 μg/d)	35 d	Decreased expression of Nrf2 in the medium and high dose groups (7 and 70 μg/d); Inflammatory reaction in testicular tissue—increased factor IL-1β (7 and 70 μg/d); Decrease in number of viable epididymis (70 μg/d); Destroyed testis tissue structure (0.7, 7, and 70 μg/d)	[3]
	Mice	PS	0.5, 4, and 10 μm	Spheres	1 mg/mL (1 mg/d)	28 d	Inflammatory reaction in testis—increased factors TNF-α and IL-6 (1 mg/d); Decreased testosterone level (1 mg/d); Abnormal sperm morphology (1 mg/d); Decreased consumption of food by tested animals (1 mg/d)	[4]
	Mice	PS	5–5.9 μm	Spheres	0.01, 0.1, 1, and 100 mg/d	42 d	Activation of p38 MAPK (0.01, 0.1, 1, and 100 mg/d); Increased level of Casp-3, TNF-α, IL-1β, and IL-6 in the testicular tissue (0.01, 0.1, 1, and 100 mg/d); Decreased concentration of testosterone (0.01, 0.1, 1, and 100 mg/d); Reduced the activity of enzymes LDH and SDH (0.01, 0.1, 1, and 100 mg/d); Decreased in number of spermatogenic cells (0.01, 0.1, 1, and 100 mg/d)	[5]

PS—polystyrene; PE—polyethylene; ROS—reactive oxygen species; MDA—malondialdehyde; SOD—superoxide dismutase; GPx—glutathione peroxidase; CAT—catalase; IL-6—interleukin-6; Nrf2—nuclear factor erythroid 2-related factor 2; IL-1β—interleukin-1beta; TNF-α—tumor necrosis factor α; p38 MAPK—p38 mitogen-activated protein kinases; Casp-3—caspase-3; LDH—lactate dehydrogenase; SDH—succinate dehydrogenase; d—day.

## Data Availability

Data sharing is not applicable.

## References

[1] Kadac-Czapska K., Jutrzenka Trzebiatowska P., Knez E., Zaleska-Medynska A., Grembecka M. (2023). Microplastics in Food—A Critical Approach to Definition, Sample Preparation, and Characterisation. Food Chem..

[2] Hu M., Palić D. (2020). Micro- and Nano-Plastics Activation of Oxidative and Inflammatory Adverse Outcome Pathways. Redox Biol..

[3] Kadac-Czapska K., Knez E., Grembecka M. (2024). Food and Human Safety: The Impact of Microplastics. Crit. Rev. Food Sci. Nutr..

[4] Siddiqui S.A., Singh S., Bahmid N.A., Shyu D.J.H., Domínguez R., Lorenzo J.M., Pereira J.A.M., Câmara J.S. (2023). Polystyrene Microplastic Particles in the Food Chain: Characteristics and Toxicity—A Review. Sci. Total Environ..

[5] Jeon S., Jeon J.H., Jeong J., Kim G., Lee S., Kim S., Maruthupandy M., Lee K., Yang S.I., Cho W.-S. (2023). Size- and Oxidative Potential-Dependent Toxicity of Environmentally Relevant Expanded Polystyrene Styrofoam Microplastics to Macrophages. J. Hazard. Mater..

[6] Kadac-Czapska K., Knez E., Gierszewska M., Olewnik-Kruszkowska E., Grembecka M. (2023). Microplastics Derived from Food Packaging Waste—Their Origin and Health Risks. Materials.

[7] Celebi Sözener Z., Cevhertas L., Nadeau K., Akdis M., Akdis C.A. (2020). Environmental Factors in Epithelial Barrier Dysfunction. J. Allergy Clin. Immunol..

[8] Pironti C., Ricciardi M., Motta O., Miele Y., Proto A., Montano L. (2021). Microplastics in the Environment: Intake through the Food Web, Human Exposure and Toxicological Effects. Toxics.

[9] Leslie H.A., van Velzen M.J.M., Brandsma S.H., Vethaak A.D., Garcia-Vallejo J.J., Lamoree M.H. (2022). Discovery and Quantification of Plastic Particle Pollution in Human Blood. Environ. Int..

[10] Kinigopoulou V., Pashalidis I., Kalderis D., Anastopoulos I. (2022). Microplastics as Carriers of Inorganic and Organic Contaminants in the Environment: A Review of Recent Progress. J. Mol. Liq..

[11] Rainieri S., Barranco A. (2019). Microplastics, a Food Safety Issue?. Trends Food Sci. Technol..

[12] Chen X., Zhou S., Liu Y., Feng Z., Mu C., Zhang T. (2024). The Combined Effects of Microplastics and Bisphenol-A on the Innate Immune System Response and Intestinal Microflora of the Swimming Crab Portunus Trituberculatus. Aquat. Toxicol..

[13] Deng Y., Yan Z., Shen R., Huang Y., Ren H., Zhang Y. (2021). Enhanced Reproductive Toxicities Induced by Phthalates Contaminated Microplastics in Male Mice (*Mus musculus*). J. Hazard. Mater..

[14] Rubin A.E., Zucker I. (2022). Interactions of Microplastics and Organic Compounds in Aquatic Environments: A Case Study of Augmented Joint Toxicity. Chemosphere.

[15] Ferrante M.C., Monnolo A., Del Piano F., Mattace Raso G., Meli R. (2022). The Pressing Issue of Micro- and Nanoplastic Contamination: Profiling the Reproductive Alterations Mediated by Oxidative Stress. Antioxidants.

[16] Ding R., Ma Y., Li T., Sun M., Sun Z., Duan J. (2023). The Detrimental Effects of Micro-and Nano-Plastics on Digestive System: An Overview of Oxidative Stress-Related Adverse Outcome Pathway. Sci. Total Environ..

[17] Paul-Pont I., Lacroix C., González Fernández C., Hégaret H., Lambert C., Le Goïc N., Frère L., Cassone A.-L., Sussarellu R., Fabioux C. (2016). Exposure of Marine Mussels Mytilus Spp. to Polystyrene Microplastics: Toxicity and Influence on Fluoranthene Bioaccumulation. Environ. Pollut..

[18] Jeong C.-B., Won E.-J., Kang H.-M., Lee M.-C., Hwang D.-S., Hwang U.-K., Zhou B., Souissi S., Lee S.-J., Lee J.-S. (2016). Microplastic Size-Dependent Toxicity, Oxidative Stress Induction, and p-JNK and p-P38 Activation in the Monogonont Rotifer (*Brachionus koreanus*). Environ. Sci. Technol..

[19] Cui J., Zhang Y., Liu L., Zhang Q., Xu S., Guo M. (2023). Polystyrene Microplastics Induced Inflammation with Activating the TLR2 Signal by Excessive Accumulation of ROS in Hepatopancreas of Carp (*Cyprinus Carpio*). Ecotoxicol. Environ. Saf..

[20] Sies H., Jones D., Fink G. (2007). Oxidative Stress. Encyclopedia of Stress.

[21] Sies H. (2015). Oxidative Stress: A Concept in Redox Biology and Medicine. Redox Biol..

[22] Sies H., Berndt C., Jones D.P. (2017). Oxidative Stress. Annu. Rev. Biochem..

[23] Niki E. (2018). Oxidative Stress and Antioxidants: Distress or Eustress?. Free Radic. Biol. Med..

[24] Sies H. (2017). Hydrogen Peroxide as a Central Redox Signaling Molecule in Physiological Oxidative Stress: Oxidative Eustress. Redox Biol..

[25] Yin M., O’Neill L.A.J. (2021). The Role of the Electron Transport Chain in Immunity. FASEB J..

[26] Mazat J.-P., Devin A., Ransac S. (2020). Modelling Mitochondrial ROS Production by the Respiratory Chain. Cell. Mol. Life Sci..

[27] Nakamura T., Naguro I., Ichijo H. (2019). Iron Homeostasis and Iron-Regulated ROS in Cell Death, Senescence and Human Diseases. Biochim. Biophys. Acta (BBA)—Gen. Subj..

[28] Yan F., Yang W., Li X., Lin T., Lun Y., Lin F., Lv S., Yan G., Liu J., Shen J. (2008). A Trifunctional Enzyme with Glutathione S-Transferase, Glutathione Peroxidase and Superoxide Dismutase Activity. Biochim. Biophys. Acta (BBA)—Gen. Subj..

[29] Forman H.J., Zhang H. (2021). Targeting Oxidative Stress in Disease: Promise and Limitations of Antioxidant Therapy. Nat. Rev. Drug Discov..

[30] Brioukhanov A.L., Netrusov A.I. (2004). Catalase and Superoxide Dismutase: Distribution, Properties, and Physiological Role in Cells of Strict Anaerobes. Biochemistry.

[31] Wang N., Wang F., Gao Y., Yin P., Pan C., Liu W., Zhou Z., Wang J. (2016). Curcumin Protects Human Adipose-Derived Mesenchymal Stem Cells against Oxidative Stress-Induced Inhibition of Osteogenesis. J. Pharmacol. Sci..

[32] Saleem U., Sabir S., Niazi S.G., Naeem M., Ahmad B. (2020). Role of Oxidative Stress and Antioxidant Defense Biomarkers in Neurodegenerative Diseases. Crit. Rev. Eukaryot. Gene Expr..

[33] He Y., Li Z., Xu T., Luo D., Chi Q., Zhang Y., Li S. (2022). Polystyrene Nanoplastics Deteriorate LPS-Modulated Duodenal Permeability and Inflammation in Mice via ROS Drived-NF-ΚB/NLRP3 Pathway. Chemosphere.

[34] Li Z., Chang X., Hu M., Fang J.K.-H., Sokolova I.M., Huang W., Xu E.G., Wang Y. (2022). Is Microplastic an Oxidative Stressor? Evidence from a Meta-Analysis on Bivalves. J. Hazard. Mater..

[35] Prata J.C., da Costa J.P., Lopes I., Duarte A.C., Rocha-Santos T. (2020). Environmental Exposure to Microplastics: An Overview on Possible Human Health Effects. Sci. Total Environ..

[36] Zhang Y., Yin K., Wang D., Wang Y., Lu H., Zhao H., Xing M. (2022). Polystyrene Microplastics-Induced Cardiotoxicity in Chickens via the ROS-Driven NF-ΚB-NLRP3-GSDMD and AMPK-PGC-1α Axes. Sci. Total Environ..

[37] Zou H., Qu H., Bian Y., Sun J., Wang T., Ma Y., Yuan Y., Gu J., Bian J., Liu Z. (2023). Polystyrene Microplastics Induce Oxidative Stress in Mouse Hepatocytes in Relation to Their Size. Int. J. Mol. Sci..

[38] Lei L., Liu M., Song Y., Lu S., Hu J., Cao C., Xie B., Shi H., He D. (2018). Polystyrene (Nano)Microplastics Cause Size-Dependent Neurotoxicity, Oxidative Damage and Other Adverse Effects in *Caenorhabditis elegans*. Environ. Sci. Nano.

[39] Tidjani A. (2000). Comparison of Formation of Oxidation Products during Photo-Oxidation of Linear Low Density Polyethylene under Different Natural and Accelerated Weathering Conditions. Polym. Degrad. Stab..

[40] Gillen K.T., Bernstein R., Celina M. (2005). Non-Arrhenius Behavior for Oxidative Degradation of Chlorosulfonated Polyethylene Materials. Polym. Degrad. Stab..

[41] Yousif E., Haddad R. (2013). Photodegradation and Photostabilization of Polymers, Especially Polystyrene: Review. Springerplus.

[42] Qiao R., Sheng C., Lu Y., Zhang Y., Ren H., Lemos B. (2019). Microplastics Induce Intestinal Inflammation, Oxidative Stress, and Disorders of Metabolome and Microbiome in Zebrafish. Sci. Total Environ..

[43] von Moos N., Burkhardt-Holm P., Köhler A. (2012). Uptake and Effects of Microplastics on Cells and Tissue of the Blue Mussel *Mytilus edulis* L. after an Experimental Exposure. Environ. Sci. Technol..

[44] Geys J., Coenegrachts L., Vercammen J., Engelborghs Y., Nemmar A., Nemery B., Hoet P.H.M. (2006). In Vitro Study of the Pulmonary Translocation of Nanoparticles. Toxicol. Lett..

[45] Lv L., Yan X., Feng L., Jiang S., Lu Z., Xie H., Sun S., Chen J., Li C. (2021). Challenge for the Detection of Microplastics in the Environment. Water Environ. Res..

[46] An R., Wang X., Yang L., Zhang J., Wang N., Xu F., Hou Y., Zhang H., Zhang L. (2021). Polystyrene Microplastics Cause Granulosa Cells Apoptosis and Fibrosis in Ovary through Oxidative Stress in Rats. Toxicology.

[47] Xie X., Deng T., Duan J., Xie J., Yuan J., Chen M. (2020). Exposure to Polystyrene Microplastics Causes Reproductive Toxicity through Oxidative Stress and Activation of the P38 MAPK Signaling Pathway. Ecotoxicol. Environ. Saf..

[48] Jeong C.-B., Kang H.-M., Lee M.-C., Kim D.-H., Han J., Hwang D.-S., Souissi S., Lee S.-J., Shin K.-H., Park H.G. (2017). Adverse Effects of Microplastics and Oxidative Stress-Induced MAPK/Nrf2 Pathway-Mediated Defense Mechanisms in the Marine Copepod Paracyclopina Nana. Sci. Rep..

[49] Lu Y., Zhang Y., Deng Y., Jiang W., Zhao Y., Geng J., Ding L., Ren H. (2016). Uptake and Accumulation of Polystyrene Microplastics in Zebrafish (Danio Rerio) and Toxic Effects in Liver. Environ. Sci. Technol..

[50] Wan Z., Wang C., Zhou J., Shen M., Wang X., Fu Z., Jin Y. (2019). Effects of Polystyrene Microplastics on the Composition of the Microbiome and Metabolism in Larval Zebrafish. Chemosphere.

[51] McCubrey J.A., LaHair M.M., Franklin R.A. (2006). Reactive Oxygen Species-Induced Activation of the MAP Kinase Signaling Pathways. Antioxid. Redox Signal.

[52] Shi X., Zhou B. (2010). The Role of Nrf2 and MAPK Pathways in PFOS-Induced Oxidative Stress in Zebrafish Embryos. Toxicol. Sci..

[53] Yu P., Liu Z., Wu D., Chen M., Lv W., Zhao Y. (2018). Accumulation of Polystyrene Microplastics in Juvenile Eriocheir Sinensis and Oxidative Stress Effects in the Liver. Aquat. Toxicol..

[54] Fleury J.-B., Baulin V.A. (2021). Microplastics Destabilize Lipid Membranes by Mechanical Stretching. Proc. Natl. Acad. Sci. USA.

[55] Nam T.-G. (2011). Lipid Peroxidation and Its Toxicological Implications. Toxicol. Res..

[56] Barboza L.G.A., Vieira L.R., Branco V., Figueiredo N., Carvalho F., Carvalho C., Guilhermino L. (2018). Microplastics Cause Neurotoxicity, Oxidative Damage and Energy-Related Changes and Interact with the Bioaccumulation of Mercury in the European Seabass, Dicentrarchus Labrax (Linnaeus, 1758). Aquat. Toxicol..

[57] Montalbetti E., Isa V., Vencato S., Louis Y., Montano S., Lavorano S., Maggioni D., Galli P., Seveso D. (2022). Short-Term Microplastic Exposure Triggers Cellular Damage through Oxidative Stress in the Soft Coral *Coelogorgia palmosa*. Mar. Biol. Res..

[58] Sun T., Zhan J., Li F., Ji C., Wu H. (2021). Evidence-Based Meta-Analysis of the Genotoxicity Induced by Microplastics in Aquatic Organisms at Environmentally Relevant Concentrations. Sci. Total Environ..

[59] Maity S., Guchhait R., De S., Pramanick K. (2023). High Doses of Nano-Polystyrene Aggravate the Oxidative Stress, DNA Damage, and the Cell Death in Onions. Environ. Pollut..

[60] Shen R., Yang K., Cheng X., Guo C., Xing X., Sun H., Liu D., Liu X., Wang D. (2022). Accumulation of Polystyrene Microplastics Induces Liver Fibrosis by Activating CGAS/STING Pathway. Environ. Pollut..

[61] He T., Qu Y., Yang X., Liu L., Xiong F., Wang D., Liu M., Sun R. (2023). Research Progress on the Cellular Toxicity Caused by Microplastics and Nanoplastics. J. Appl. Toxicol..

[62] Malinowska K., Bukowska B., Piwoński I., Foksiński M., Kisielewska A., Zarakowska E., Gackowski D., Sicińska P. (2022). Polystyrene Nanoparticles: The Mechanism of Their Genotoxicity in Human Peripheral Blood Mononuclear Cells. Nanotoxicology.

[63] Çobanoğlu H., Belivermiş M., Sıkdokur E., Kılıç Ö., Çayır A. (2021). Genotoxic and Cytotoxic Effects of Polyethylene Microplastics on Human Peripheral Blood Lymphocytes. Chemosphere.

[64] Lu K., Qiao R., An H., Zhang Y. (2018). Influence of Microplastics on the Accumulation and Chronic Toxic Effects of Cadmium in Zebrafish (Danio Rerio). Chemosphere.

[65] Ribeiro F., Garcia A.R., Pereira B.P., Fonseca M., Mestre N.C., Fonseca T.G., Ilharco L.M., Bebianno M.J. (2017). Microplastics Effects in Scrobicularia Plana. Mar. Pollut. Bull..

[66] Avio C.G., Gorbi S., Milan M., Benedetti M., Fattorini D., D’Errico G., Pauletto M., Bargelloni L., Regoli F. (2015). Pollutants Bioavailability and Toxicological Risk from Microplastics to Marine Mussels. Environ. Pollut..

[67] Hamed M., Soliman H.A.M., Osman A.G.M., Sayed A.E.-D.H. (2020). Antioxidants and Molecular Damage in Nile Tilapia (Oreochromis Niloticus) after Exposure to Microplastics. Environ. Sci. Pollut. Res..

[68] Bahadur P., Maharjan A., Acharya M., Lee D., Kusma S., Gautam R., Kwon J.-T., Kim C., Kim K., Kim H. (2023). Polytetrafluorethylene Microplastic Particles Mediated Oxidative Stress, Inflammation, and Intracellular Signaling Pathway Alteration in Human Derived Cell Lines. Sci. Total Environ..

[69] Boháčková J., Havlíčková L., Semerád J., Titov I., Trhlíková O., Beneš H., Cajthaml T. (2023). In Vitro Toxicity Assessment of Polyethylene Terephthalate and Polyvinyl Chloride Microplastics Using Three Cell Lines from Rainbow Trout (Oncorhynchus Mykiss). Chemosphere.

[70] Liu T., Hou B., Wang Z., Yang Y. (2022). Polystyrene Microplastics Induce Mitochondrial Damage in Mouse GC-2 Cells. Ecotoxicol. Environ. Saf..

[71] Wang Q., Bai J., Ning B., Fan L., Sun T., Fang Y., Wu J., Li S., Duan C., Zhang Y. (2020). Effects of Bisphenol A and Nanoscale and Microscale Polystyrene Plastic Exposure on Particle Uptake and Toxicity in Human Caco-2 Cells. Chemosphere.

[72] Zhou Y., Wu Q., Li Y., Feng Y., Wang Y., Cheng W. (2023). Low-Dose of Polystyrene Microplastics Induce Cardiotoxicity in Mice and Human-Originated Cardiac Organoids. Environ. Int..

[73] Paget V., Dekali S., Kortulewski T., Grall R., Gamez C., Blazy K., Aguerre-Chariol O., Chevillard S., Braun A., Rat P. (2015). Specific Uptake and Genotoxicity Induced by Polystyrene Nanobeads with Distinct Surface Chemistry on Human Lung Epithelial Cells and Macrophages. PLoS ONE.

[74] Schmidt A., da Silva Brito W.A., Singer D., Mühl M., Berner J., Saadati F., Wolff C., Miebach L., Wende K., Bekeschus S. (2023). Short- and Long-Term Polystyrene Nano- and Microplastic Exposure Promotes Oxidative Stress and Divergently Affects Skin Cell Architecture and Wnt/Beta-Catenin Signaling. Part. Fibre Toxicol..

[75] Magara G., Khan F.R., Pinti M., Syberg K., Inzirillo A., Elia A.C. (2019). Effects of Combined Exposures of Fluoranthene and Polyethylene or Polyhydroxybutyrate Microplastics on Oxidative Stress Biomarkers in the Blue Mussel (*Mytilus edulis*). J. Toxicol. Environ. Health A.

[76] Chen Wongworawat Y., Filippova M., Williams V.M., Filippov V., Duerksen-Hughes P.J. (2016). Chronic Oxidative Stress Increases the Integration Frequency of Foreign DNA and Human Papillomavirus 16 in Human Keratinocytes. Am. J. Cancer Res..

[77] Cohen S. (2016). Reactive Oxygen Species and Serous Epithelial Ovarian Adenocarcinoma. Cancer Res. J..

[78] Ubezio P., Civoli F. (1994). Flow Cytometric Detection of Hydrogen Peroxide Production Induced by Doxorubicin in Cancer Cells. Free Radic. Biol. Med..

[79] Peshavariya H.M., Dusting G.J., Selemidis S. (2007). Analysis of Dihydroethidium Fluorescence for the Detection of Intracellular and Extracellular Superoxide Produced by NADPH Oxidase. Free Radic. Res..

[80] Trotti R., Carratelli M., Barbieri M. (2002). Performance and Clinical Application of a New, Fast Method for the Detection of Hydroperoxides in Serum. Panminerva Med..

[81] Katerji M., Filippova M., Duerksen-Hughes P. (2019). Approaches and Methods to Measure Oxidative Stress in Clinical Samples: Research Applications in the Cancer Field. Oxid. Med. Cell Longev..

[82] Buwono N.R., Risjani Y., Soegianto A. (2022). Oxidative Stress Responses of Microplastic-Contaminated Gambusia Affinis Obtained from the Brantas River in East Java, Indonesia. Chemosphere.

[83] Giustarini D., Dalle-Donne I., Tsikas D., Rossi R. (2009). Oxidative Stress and Human Diseases: Origin, Link, Measurement, Mechanisms, and Biomarkers. Crit. Rev. Clin. Lab. Sci..

[84] Bernardes S.S., de Souza-Neto F.P., Ramalho L.N.Z., Derossi D.R., Guarnier F.A., da Silva C.F.N., Melo G.P., Simão A.N.C., Cecchini R., Cecchini A.L. (2015). Systemic Oxidative Profile after Tumor Removal and the Tumor Microenvironment in Melanoma Patients. Cancer Lett..

[85] Blasi M.A., Maresca V., Roccella M., Roccella F., Sansolini T., Grammatico P., Balestrazzi E., Picardo M. (1999). Antioxidant Pattern in Uveal Melanocytes and Melanoma Cell Cultures. Investig. Ophthalmol. Vis. Sci..

[86] Yu R., Zhao G., Christman J.W., Xiao L., van Breemen R.B. (2013). Method Development and Validation for Ultra-High Pressure Liquid Chromatography/Tandem Mass Spectrometry Determination of Multiple Prostanoids in Biological Samples. J. AOAC Int..

[87] Jo M., Nishikawa T., Nakajima T., Okada Y., Yamaguchi K., Mitsuyoshi H., Yasui K., Minami M., Iwai M., Kagawa K. (2011). Oxidative Stress Is Closely Associated with Tumor Angiogenesis of Hepatocellular Carcinoma. J. Gastroenterol..

[88] Skrzydlewska E. (2005). Lipid Peroxidation and Antioxidant Status in Colorectal Cancer. World J. Gastroenterol..

[89] Facundo H.T.F., Brandt C.T., Owen J.S., Lima V.L.M. (2004). Elevated Levels of Erythrocyte-Conjugated Dienes Indicate Increased Lipid Peroxidation in Schistosomiasis Mansoni Patients. Braz. J. Med. Biol. Res..

[90] Nouroozzadeh J., Tajaddinisarmadi J., Wolff S.P. (1994). Measurement of Plasma Hydroperoxide Concentrations by the Ferrous Oxidation-Xylenol Orange Assay in Conjunction with Triphenylphosphine. Anal. Biochem..

[91] Gonzalez-Hunt C.P., Wadhwa M., Sanders L.H. (2018). DNA Damage by Oxidative Stress: Measurement Strategies for Two Genomes. Curr. Opin. Toxicol..

[92] Cadet J., Davies K.J.A., Medeiros M.H., Di Mascio P., Wagner J.R. (2017). Formation and Repair of Oxidatively Generated Damage in Cellular DNA. Free Radic. Biol. Med..

[93] Anson R.M., Mason P.A., Bohr V.A. (2006). Gene-Specific and Mitochondrial Repair of Oxidative DNA Damage. Methods Mol. Biol..

[94] Collins A.R., Cadet J., Mőller L., Poulsen H.E., Viña J. (2004). Are We Sure We Know How to Measure 8-Oxo-7,8-Dihydroguanine in DNA from Human Cells?. Arch. Biochem. Biophys..

[95] Singh N.P., McCoy M.T., Tice R.R., Schneider E.L. (1988). A Simple Technique for Quantitation of Low Levels of DNA Damage in Individual Cells. Exp. Cell Res..

[96] Sun S., Shi W., Tang Y., Han Y., Du X., Zhou W., Zhang W., Sun C., Liu G. (2021). The Toxic Impacts of Microplastics (MPs) and Polycyclic Aromatic Hydrocarbons (PAHs) on Haematic Parameters in a Marine Bivalve Species and Their Potential Mechanisms of Action. Sci. Total Environ..

[97] Lin W., Jiang R., Hu S., Xiao X., Wu J., Wei S., Xiong Y., Ouyang G. (2019). Investigating the Toxicities of Different Functionalized Polystyrene Nanoplastics on Daphnia Magna. Ecotoxicol. Environ. Saf..

[98] Christen V., Camenzind M., Fent K. (2014). Silica Nanoparticles Induce Endoplasmic Reticulum Stress Response, Oxidative Stress and Activate the Mitogen-Activated Protein Kinase (MAPK) Signaling Pathway. Toxicol. Rep..

[99] Paraiso K.H.T., Van Der Kooi K., Messina J.L., Smalley K.S.M. (2010). Measurement of Constitutive MAPK and PI3K/AKT Signaling Activity in Human Cancer Cell Lines. Methods Enzymol..

[100] Kefaloyianni E., Gaitanaki C., Beis I. (2006). ERK1/2 and P38-MAPK Signalling Pathways, through MSK1, Are Involved in NF-ΚB Transactivation during Oxidative Stress in Skeletal Myoblasts. Cell Signal.

[101] Xu B., Lang L.-M., Lian S., Guo J.-R., Wang J.-F., Yang H.-M., Li S.-Z. (2019). Oxidation Stress-Mediated MAPK Signaling Pathway Activation Induces Neuronal Loss in the CA1 and CA3 Regions of the Hippocampus of Mice Following Chronic Cold Exposure. Brain Sci..

[102] Naderi J., Hung M., Pandey S. (2003). Oxidative Stress-Induced Apoptosis in Dividing Fibroblasts Involves Activation of P38 MAP Kinase and over-Expression of Bax: Resistance of Quiescent Cells to Oxidative Stress. Apoptosis.

[103] Halliwell B., Gutteridge J.M.C. (1986). Oxygen Free Radicals and Iron in Relation to Biology and Medicine: Some Problems and Concepts. Arch. Biochem. Biophys..

[104] Misra H.P., Fridovich I. (1972). The Role of Superoxide Anion in the Autoxidation of Epinephrine and a Simple Assay for Superoxide Dismutase. J. Biol. Chem..

[105] Sun Y., Oberley L.W., Li Y. (1988). A Simple Method for Clinical Assay of Superoxide Dismutase. Clin. Chem..

[106] Djordjević V.B. (2004). Free Radicals in Cell Biology. Int. Rev. Cytol..

[107] Woźniak A., Masiak R., Szpinda M., Mila-Kierzenkowska C., Woźniak B., Makarewicz R., Szpinda A. (2012). Oxidative Stress Markers in Prostate Cancer Patients after HDR Brachytherapy Combined with External Beam Radiation. Oxid. Med. Cell Longev..

[108] Arthur J.R. (2001). The Glutathione Peroxidases. Cell. Mol. Life Sci..

[109] Güner G., İşlekel H., Oto Ö., Hazan E., Açikel Ü. (1996). Evaluation of Some Antioxidant Enzymes in Lung Carcinoma Tissue. Cancer Lett..

[110] Johansson L.H., Håkan Borg L.A. (1988). A Spectrophotometric Method for Determination of Catalase Activity in Small Tissue Samples. Anal. Biochem..

[111] Ellman G.L. (1959). Tissue Sulfhydryl Groups. Arch. Biochem. Biophys..

[112] Strzelczyk J.K., Wielkoszyński T., Krakowczyk Ł., Adamek B., Zalewska-Ziob M., Gawron K., Kasperczyk J., Wiczkowski A. (2012). The Activity of Antioxidant Enzymes in Colorectal Adenocarcinoma and Corresponding Normal Mucosa. Acta Biochim. Pol..

[113] Hubatsch I., Ridderström M., Mannervik B. (1998). Human Glutathione Transferase A4-4: An Alpha Class Enzyme with High Catalytic Efficiency in the Conjugation of 4-Hydroxynonenal and Other Genotoxic Products of Lipid Peroxidation. Biochem. J..

[114] Čapek J., Roušar T. (2021). Detection of Oxidative Stress Induced by Nanomaterials in Cells—The Roles of Reactive Oxygen Species and Glutathione. Molecules.

[115] Schirinzi G.F., Pérez-Pomeda I., Sanchís J., Rossini C., Farré M., Barceló D. (2017). Cytotoxic Effects of Commonly Used Nanomaterials and Microplastics on Cerebral and Epithelial Human Cells. Environ. Res..

[116] Cole M., Galloway T.S. (2015). Ingestion of Nanoplastics and Microplastics by Pacific Oyster Larvae. Environ. Sci. Technol..

[117] Salimi A., Alavehzadeh A., Ramezani M., Pourahmad J. (2022). Differences in Sensitivity of Human Lymphocytes and Fish Lymphocytes to Polyvinyl Chloride Microplastic Toxicity. Toxicol. Ind. Health.

[118] Bonanomi M., Salmistraro N., Porro D., Pinsino A., Colangelo A.M., Gaglio D. (2022). Polystyrene Micro and Nano-Particles Induce Metabolic Rewiring in Normal Human Colon Cells: A Risk Factor for Human Health. Chemosphere.

[119] Saenen N.D., Witters M.S., Hantoro I., Tejeda I., Ethirajan A., Van Belleghem F., Smeets K. (2023). Polystyrene Microplastics of Varying Sizes and Shapes Induce Distinct Redox and Mitochondrial Stress Responses in a Caco-2 Monolayer. Antioxidants.

[120] Wu B., Wu X., Liu S., Wang Z., Chen L. (2019). Size-Dependent Effects of Polystyrene Microplastics on Cytotoxicity and Efflux Pump Inhibition in Human Caco-2 cells. Chemosphere.

[121] Zhang Y., Wang S., Olga V., Xue Y., Lv S., Diao X., Zhang Y., Han Q., Zhou H. (2022). The Potential Effects of Microplastic Pollution on Human Digestive Tract Cells. Chemosphere.

[122] Chen Y.-C., Chen K.-F., Lin K.-Y.A., Chen J.-K., Jiang X.-Y., Lin C.-H. (2022). The Nephrotoxic Potential of Polystyrene Microplastics at Realistic Environmental Concentrations. J. Hazard. Mater..

[123] Visalli G., Facciolà A., Pruiti Ciarello M., De Marco G., Maisano M., Di Pietro A. (2021). Acute and Sub-Chronic Effects of Microplastics (3 and 10 Μm) on the Human Intestinal Cells HT-29. Int. J. Environ. Res. Public Health.

[124] Florance I., Chandrasekaran N., Gopinath P.M., Mukherjee A. (2022). Exposure to Polystyrene Nanoplastics Impairs Lipid Metabolism in Human and Murine Macrophages in Vitro. Ecotoxicol. Environ. Saf..

[125] Watson H. (2015). Biological Membranes. Essays Biochem..

[126] Liu L., Xu K., Zhang B., Ye Y., Zhang Q., Jiang W. (2021). Cellular Internalization and Release of Polystyrene Microplastics and Nanoplastics. Sci. Total Environ..

[127] Monnery B.D., Wright M., Cavill R., Hoogenboom R., Shaunak S., Steinke J.H.G., Thanou M. (2017). Cytotoxicity of Polycations: Relationship of Molecular Weight and the Hydrolytic Theory of the Mechanism of Toxicity. Int. J. Pharm..

[128] Wang W., Zhang J., Qiu Z., Cui Z., Li N., Li X., Wang Y., Zhang H., Zhao C. (2022). Effects of Polyethylene Microplastics on Cell Membranes: A Combined Study of Experiments and Molecular Dynamics Simulations. J. Hazard. Mater..

[129] Jia R., Han J., Liu X., Li K., Lai W., Bian L., Yan J., Xi Z. (2023). Exposure to Polypropylene Microplastics via Oral Ingestion Induces Colonic Apoptosis and Intestinal Barrier Damage through Oxidative Stress and Inflammation in Mice. Toxics.

[130] Lawrence R.E., Zoncu R. (2019). The Lysosome as a Cellular Centre for Signalling, Metabolism and Quality Control. Nat. Cell Biol..

[131] Deng J., Ibrahim M.S., Tan L.Y., Yeo X.Y., Lee Y.A., Park S.J., Wüstefeld T., Park J.W., Jung S., Cho N.J. (2022). Microplastics Released from Food Containers Can Suppress Lysosomal Activity in Mouse Macrophages. J. Hazard. Mater..

[132] Fiorentino I., Gualtieri R., Barbato V., Mollo V., Braun S., Angrisani A., Turano M., Furia M., Netti P.A., Guarnieri D. (2015). Energy Independent Uptake and Release of Polystyrene Nanoparticles in Primary Mammalian Cell Cultures. Exp. Cell Res..

[133] Florance I., Ramasubbu S., Mukherjee A., Chandrasekaran N. (2021). Polystyrene Nanoplastics Dysregulate Lipid Metabolism in Murine Macrophages in Vitro. Toxicology.

[134] Canesi L., Ciacci C., Fabbri R., Balbi T., Salis A., Damonte G., Cortese K., Caratto V., Monopoli M.P., Dawson K. (2016). Interactions of Cationic Polystyrene Nanoparticles with Marine Bivalve Hemocytes in a Physiological Environment: Role of Soluble Hemolymph Proteins. Environ. Res..

[135] Wang F., Bexiga M.G., Anguissola S., Boya P., Simpson J.C., Salvati A., Dawson K.A. (2013). Time Resolved Study of Cell Death Mechanisms Induced by Amine-Modified Polystyrene Nanoparticles. Nanoscale.

[136] Golpich M., Amini E., Mohamed Z., Azman Ali R., Mohamed Ibrahim N., Ahmadiani A. (2017). Mitochondrial Dysfunction and Biogenesis in Neurodegenerative Diseases: Pathogenesis and Treatment. CNS Neurosci. Ther..

[137] Wallace D.C. (2005). A Mitochondrial Paradigm of Metabolic and Degenerative Diseases, Aging, and Cancer: A Dawn for Evolutionary Medicine. Annu. Rev. Genet..

[138] Wei J., Wang X., Liu Q., Zhou N., Zhu S., Li Z., Li X., Yao J., Zhang L. (2021). The Impact of Polystyrene Microplastics on Cardiomyocytes Pyroptosis through NLRP3/Caspase-1 Signaling Pathway and Oxidative Stress in Wistar Rats. Environ. Toxicol..

[139] Zhang W., Sun X., Qi X., Liu X., Zhang Y., Qiao S., Lin H. (2022). Di-(2-Ethylhexyl) Phthalate and Microplastics Induced Neuronal Apoptosis through the PI3K/AKT Pathway and Mitochondrial Dysfunction. J. Agric. Food Chem..

[140] Zorov D.B., Juhaszova M., Sollott S.J. (2006). Mitochondrial ROS-Induced ROS Release: An Update and Review. Biochim. Biophys. Acta (BBA)—Bioenerg..

[141] Lee S.E., Yi Y., Moon S., Yoon H., Park Y.S. (2022). Impact of Micro- and Nanoplastics on Mitochondria. Metabolites.

[142] Malinowska K., Sicińska P., Michałowicz J., Bukowska B. (2023). The Effects of Non-Functionalized Polystyrene Nanoparticles of Different Diameters on the Induction of Apoptosis and MTOR Level in Human Peripheral Blood Mononuclear Cells. Chemosphere.

[143] Félix L., Carreira P., Peixoto F. (2023). Effects of Chronic Exposure of Naturally Weathered Microplastics on Oxidative Stress Level, Behaviour, and Mitochondrial Function of Adult Zebrafish (Danio Rerio). Chemosphere.

[144] Herrala M., Huovinen M., Järvelä E., Hellman J., Tolonen P., Lahtela-Kakkonen M., Rysä J. (2023). Micro-Sized Polyethylene Particles Affect Cell Viability and Oxidative Stress Responses in Human Colorectal Adenocarcinoma Caco-2 and HT-29 Cells. Sci. Total Environ..

[145] Pan L., Yu D., Zhang Y., Zhu C., Yin Q., Hu Y., Zhang X., Yue R., Xiong X. (2021). Polystyrene Microplastics-Triggered Mitophagy and Oxidative Burst via Activation of PERK Pathway. Sci. Total Environ..

[146] Almanza A., Carlesso A., Chintha C., Creedican S., Doultsinos D., Leuzzi B., Luís A., McCarthy N., Montibeller L., More S. (2019). Endoplasmic Reticulum Stress Signalling—From Basic Mechanisms to Clinical Applications. FEBS J..

[147] Haeri M., Knox B.E. (2012). Endoplasmic Reticulum Stress and Unfolded Protein Response Pathways: Potential for Treating Age-Related Retinal Degeneration. J. Ophthalmic Vis. Res..

[148] Wang W., Guan J., Feng Y., Nie L., Xu Y., Xu H., Fu F. (2022). Polystyrene Microplastics Induced Nephrotoxicity Associated with Oxidative Stress, Inflammation, and Endoplasmic Reticulum Stress in Juvenile Rats. Front. Nutr..

[149] Wang F., Zhang Q., Cui J., Bao B., Deng X., Liu L., Guo M. (2023). Polystyrene Microplastics Induce Endoplasmic Reticulum Stress, Apoptosis and Inflammation by Disrupting the Gut Microbiota in Carp Intestines. Environ. Pollut..

[150] Wang W., Guan J., Feng Y., Liu S., Zhao Y., Xu Y., Xu H., Fu F. (2023). Polystyrene Microplastics Induced Ovarian Toxicity in Juvenile Rats Associated with Oxidative Stress and Activation of the PERK-EIF2α-ATF4-CHOP Signaling Pathway. Toxics.

[151] Saemi-Komsari M., Pashaei R., Abbasi S., Esmaeili H.R., Dzingelevičienė R., Shirkavand Hadavand B., Pasalari Kalako M., Szultka-Mlynska M., Gadzała-Kopciuch R., Buszewski B. (2023). Accumulation of Polystyrene Nanoplastics and Triclosan by a Model Tooth-Carp Fish, *Aphaniops hormuzensis* (Teleostei: Aphaniidae). Environ. Pollut..

[152] da Silva Brito W.A., Mutter F., Wende K., Cecchini A.L., Schmidt A., Bekeschus S. (2022). Consequences of Nano and Microplastic Exposure in Rodent Models: The Known and Unknown. Part. Fibre Toxicol..

[153] Osman A.I., Hosny M., Eltaweil A.S., Omar S., Elgarahy A.M., Farghali M., Yap P.-S., Wu Y.-S., Nagandran S., Batumalaie K. (2023). Microplastic Sources, Formation, Toxicity and Remediation: A Review. Environ. Chem. Lett..

[154] Bhuyan M.S. (2022). Effects of Microplastics on Fish and in Human Health. Front. Environ. Sci..

[155] Espinosa C., García Beltrán J.M., Esteban M.A., Cuesta A. (2018). In Vitro Effects of Virgin Microplastics on Fish Head-Kidney Leucocyte Activities. Environ. Pollut..

[156] Li X., Zhang T., Lv W., Wang H., Chen H., Xu Q., Cai H., Dai J. (2022). Intratracheal Administration of Polystyrene Microplastics Induces Pulmonary Fibrosis by Activating Oxidative Stress and Wnt/β-Catenin Signaling Pathway in Mice. Ecotoxicol. Environ. Saf..

[157] Zeng Y., Deng B., Kang Z., Araujo P., Mjøs S.A., Liu R., Lin J., Yang T., Qu Y. (2023). Tissue Accumulation of Polystyrene Microplastics Causes Oxidative Stress, Hepatopancreatic Injury and Metabolome Alterations in *Litopenaeus vannamei*. Ecotoxicol. Environ. Saf..

[158] Deng Y., Zhang Y., Lemos B., Ren H. (2017). Tissue Accumulation of Microplastics in Mice and Biomarker Responses Suggest Widespread Health Risks of Exposure. Sci. Rep..

[159] Sadasivam N., Kim Y.-J., Radhakrishnan K., Kim D.-K. (2022). Oxidative Stress, Genomic Integrity, and Liver Diseases. Molecules.

[160] Yang B.Z., Huang H. (2019). Effect of Microplastics on Antioxidant Enzyme System in Juvenile Red Crucian Carp. Environ. Sci. Technol. China.

[161] Jeong J., Choi J. (2019). Adverse Outcome Pathways Potentially Related to Hazard Identification of Microplastics Based on Toxicity Mechanisms. Chemosphere.

[162] Peters A.E., Mihalas B.P., Bromfield E.G., Roman S.D., Nixon B., Sutherland J.M. (2020). Autophagy in Female Fertility: A Role in Oxidative Stress and Aging. Antioxid. Redox Signal.

[163] Silva E., Almeida H., Castro J.P. (2020). (In)Fertility and Oxidative Stress: New Insights into Novel Redox Mechanisms Controlling Fundamental Reproductive Processes. Oxid. Med. Cell Longev..

[164] Jeyavani J., Sibiya A., Bhavaniramya S., Mahboob S., Al-Ghanim K.A., Nisa Z., Riaz M.N., Nicoletti M., Govindarajan M., Vaseeharan B. (2022). Toxicity Evaluation of Polypropylene Microplastic on Marine Microcrustacean Artemia Salina: An Analysis of Implications and Vulnerability. Chemosphere.

[165] Wegner A., Besseling E., Foekema E.M., Kamermans P., Koelmans A.A. (2012). Effects of Nanopolystyrene on the Feeding Behavior of the Blue Mussel (*Mytilus edulis* L.). Environ. Toxicol. Chem..

[166] Zhang C., Chen X., Wang J., Tan L. (2017). Toxic Effects of Microplastic on Marine Microalgae Skeletonema Costatum: Interactions between Microplastic and Algae. Environ. Pollut..

[167] Agarwal A., Parekh N., Panner Selvam M.K., Henkel R., Shah R., Homa S.T., Ramasamy R., Ko E., Tremellen K., Esteves S. (2019). Male Oxidative Stress Infertility (MOSI): Proposed Terminology and Clinical Practice Guidelines for Management of Idiopathic Male Infertility. World J. Mens. Health.

[168] Agarwal A., Gupta S., Sharma R.K. (2005). Role of Oxidative Stress in Female Reproduction. Reprod. Biol. Endocrinol..

[169] Kim K., Yoon H., Choi J.S., Jung Y.-J., Park J.-W. (2022). Chronic Effects of Nano and Microplastics on Reproduction and Development of Marine Copepod Tigriopus Japonicus. Ecotoxicol. Environ. Saf..

[170] Hou B., Wang F., Liu T., Wang Z. (2021). Reproductive Toxicity of Polystyrene Microplastics: In Vivo Experimental Study on Testicular Toxicity in Mice. J. Hazard. Mater..

[171] Jin H., Ma T., Sha X., Liu Z., Zhou Y., Meng X., Chen Y., Han X., Ding J. (2021). Polystyrene Microplastics Induced Male Reproductive Toxicity in Mice. J. Hazard. Mater..

[172] Liu Z., Zhuan Q., Zhang L., Meng L., Fu X., Hou Y. (2022). Polystyrene Microplastics Induced Female Reproductive Toxicity in Mice. J. Hazard. Mater..

[173] Li Y., Ye Y., Rihan N., Jiang Q., Liu X., Zhao Y., Che X. (2023). Polystyrene Nanoplastics Decrease Nutrient Accumulation, Disturb Sex Hormones, and Inhibit Reproductive Development in Juvenile Macrobrachium Nipponense. Sci. Total Environ..

[174] Jaafarzadeh Haghighi Fard N., Mohammadi M.J., Jahedi F. (2023). Effects of Nano and Microplastics on the Reproduction System: In Vitro and in Vivo Studies Review. Food Chem. Toxicol..

[175] Wei Z., Wang Y., Wang S., Xie J., Han Q., Chen M. (2022). Comparing the Effects of Polystyrene Microplastics Exposure on Reproduction and Fertility in Male and Female Mice. Toxicology.

[176] Zhou Y., Zhang C.-Y., Duan J.-X., Li Q., Yang H.-H., Sun C.-C., Zhang J., Luo X.-Q., Liu S.-K. (2020). Vasoactive Intestinal Peptide Suppresses the NLRP3 Inflammasome Activation in Lipopolysaccharide-Induced Acute Lung Injury Mice and Macrophages. Biomed. Pharmacother..

[177] Wang J., Li Y., Lu L., Zheng M., Zhang X., Tian H., Wang W., Ru S. (2019). Polystyrene Microplastics Cause Tissue Damages, Sex-Specific Reproductive Disruption and Transgenerational Effects in Marine Medaka (*Oryzias melastigma*). Environ. Pollut..

[178] Qiang L., Cheng J. (2021). Exposure to Polystyrene Microplastics Impairs Gonads of Zebrafish (*Danio rerio*). Chemosphere.

[179] Liu Y., Zhang J., Zhao H., Cai J., Sultan Y., Fang H., Zhang B., Ma J. (2022). Effects of Polyvinyl Chloride Microplastics on Reproduction, Oxidative Stress and Reproduction and Detoxification-Related Genes in Daphnia Magna. Comp. Biochem. Physiol. Part C Toxicol. Pharmacol..

[180] Nobre C.R., Santana M.F.M., Maluf A., Cortez F.S., Cesar A., Pereira C.D.S., Turra A. (2015). Assessment of Microplastic Toxicity to Embryonic Development of the Sea Urchin *Lytechinus variegatus* (Echinodermata: Echinoidea). Mar. Pollut. Bull..

[181] Afreen V., Hashmi K., Nasir R., Saleem A., Khan M.I., Akhtar M.F. (2023). Adverse Health Effects and Mechanisms of Microplastics on Female Reproductive System: A Descriptive Review. Environ. Sci. Pollut. Res..

[182] Senathirajah K., Attwood S., Bhagwat G., Carbery M., Wilson S., Palanisami T. (2021). Estimation of the Mass of Microplastics Ingested—A Pivotal First Step towards Human Health Risk Assessment. J. Hazard. Mater..

[183] Pletz M. (2022). Ingested Microplastics: Do Humans Eat One Credit Card per Week?. J. Hazard. Mater. Lett..

[184] Mohamed Nor N.H., Kooi M., Diepens N.J., Koelmans A.A. (2021). Lifetime Accumulation of Microplastic in Children and Adults. Environ. Sci. Technol..

[185] Jones N.R., de Jersey A.M., Lavers J.L., Rodemann T., Rivers-Auty J. (2024). Identifying Laboratory Sources of Microplastic and Nanoplastic Contamination from the Air, Water, and Consumables. J. Hazard. Mater..

[186] Bogdanowicz A., Zubrowska-Sudol M., Krasinski A., Sudol M. (2021). Cross-Contamination as a Problem in Collection and Analysis of Environmental Samples Containing Microplastics—A Review. Sustainability.

[187] Jeyavani J., Sibiya A., Gopi N., Mahboob S., Riaz M.N., Vaseeharan B. (2022). Dietary Consumption of Polypropylene Microplastics Alter the Biochemical Parameters and Histological Response in Freshwater Benthic Mollusc *Pomacea paludosa*. Environ. Res..

[188] Xing Y., Zhu X., Duan Y., Huang J., Nan Y., Zhang J. (2023). Toxic Effects of Nitrite and Microplastics Stress on Histology, Oxidative Stress, and Metabolic Function in the Gills of Pacific White Shrimp, *Litopenaeus vannamei*. Mar. Pollut. Bull..

[189] Yedier S., Yalçınkaya S.K., Bostancı D. (2023). Exposure to Polypropylene Microplastics via Diet and Water Induces Oxidative Stress in Cyprinus Carpio. Aquat. Toxicol..

[190] Kelpsiene E., Ekvall M.T., Lundqvist M., Torstensson O., Hua J., Cedervall T. (2022). Review of Ecotoxicological Studies of Widely Used Polystyrene Nanoparticles. Environ. Sci. Process Impacts.

[191] Danso D., Chow J., Streit W.R. (2019). Plastics: Environmental and Biotechnological Perspectives on Microbial Degradation. Appl. Environ. Microbiol..

[192] Li Y., Tao L., Wang Q., Wang F., Li G., Song M. (2023). Potential Health Impact of Microplastics: A Review of Environmental Distribution, Human Exposure, and Toxic Effects. Environ. Health.

[193] Oßmann B.E., Sarau G., Holtmannspötter H., Pischetsrieder M., Christiansen S.H., Dicke W. (2018). Small-Sized Microplastics and Pigmented Particles in Bottled Mineral Water. Water Res..

[194] Haldar S., Yhome N., Muralidaran Y., Rajagopal S., Mishra P. (2023). Nanoplastics Toxicity Specific to Liver in Inducing Metabolic Dysfunction—A Comprehensive Review. Genes.

